# Accumulation of saposin in dystrophic neurites is linked to impaired lysosomal functions in Alzheimer’s disease brains

**DOI:** 10.1186/s13024-021-00464-1

**Published:** 2021-07-02

**Authors:** Md Golam Sharoar, Sarah Palko, Yingying Ge, Takaomi C. Saido, Riqiang Yan

**Affiliations:** 1 Department of Neuroscience, University of Connecticut Health, Farmington, CT 06032 USA; 2 Laboratory for Proteolytic Neuroscience, RIKEN Brain Science Institute, Wako-shi, Saitama, Japan

**Keywords:** Alzheimer’s disease, Aging, Dystrophic neurites, Lysosomes, Saposin-C, Prosaposin, ATG9, LAMP1 and galectin-3

## Abstract

Neuritic plaques in Alzheimer’s disease (AD) brains refer to β-amyloid (Aβ) plaques surrounded by dystrophic neurites (DNs), activated microglia and reactive astrocytes. Most recently, we showed that DNs form sequentially in three layers during plaque growth. Although lysosomal proteins such as LAMP1 are found in DNs, it is not clear how many and how early lysosomal proteins are involved in forming neuritic plaques. To answer this unmet question, we examined APP knock-in (APP^NL-G-F^), 5xFAD and APP/PS1ΔE9 mouse brains and found that the lysosomal activator proteins saposins (SAPs) and LAMP1 were accumulated to surround Aβ plaques at the earliest stage, namely the 1st layer of DNs. Noticeably, lysosomal hydrolases were not detectable in these early DNs, suggesting that DNs at this early stage likely enrich dysfunctional lysosomes. In old AD mouse brains and in the later stage of human AD brains, SAP-C^+^-DNs and LAMP1^+^-DNs were gradually reduced in concomitant with the growth of amyloid plaques. Remarkably, the observed LAMP1 immunoreactivity near plaques in aged AD mouse and human brains were actually associated with disease-associated microglia rather than neuronal sources, likely reflecting more severely impaired lysosomal functions in neurons. Western blot analyses showed increased levels of SAP-C in AD mouse brains, and Aβ oligomers induced elevated levels of SAP-C in cellular assays. The elevated protein levels of SAP-C in AD mouse brains during plaque growth potentially contributed lysosomal membrane leakage and loss of hydrolases. Together, our study indicates that lysosomal functions are impaired by being entrapped in DNs early during plaque growth, and this may viciously facilitate growth of amyloid plaques.

**Supplementary Information:**

The online version contains supplementary material available at 10.1186/s13024-021-00464-1.

## Introduction

Alzheimer’s disease (AD) is the most common form of aging-associated dementia and is clinically diagnosed by cognitive deficits [1]. Over the past three decades of AD research, the most widely studied “amyloid cascade hypothesis” enters on the sequential pathological changes that abnormal accumulation of β-amyloid peptides (Aβ) is the prominent early event, leading to neuronal abnormalities, development of senile (neuritic) plaques, changes in tau phosphorylation and the emergence of various cognitive symptoms [2]. Comparative studies on cognition in AD patients, as well as the presence of AD biomarkers and neuroimaging studies, demonstrate a pre-clinical phase of 10–20 years that precedes the manifestation of symptomatic AD [3–5]. Specifically, the toxic form of oligomeric Aβ during the pre-clinical phase induces local toxicity that advances both intra- and extra-neuronal pathologies such as neurofibrillary tangles, dystrophic neurites (DNs), mitochondrial degeneration, and glial dysfunction [3, 6, 7]. The accumulation of diversified proteins and organelles in DNs surrounding amyloid plaques correlates with the dysfunction of various neuronal processes during disease progression [8–15]. Because the pre-clinical stage precedes symptomatic disease onset by a decade or more [16–18], it is critical to understand exactly how and when Aβ exerts harmful effects during the development of amyloid plaques in AD brains.

One critical pathological feature in AD brains is the presence of DNs, which are recognized as swollen bulbous or ring-like neuritic processes [19–21]. DNs can be detected by antibodies specific to autophagy proteins, tubular ER proteins, ubiquitin, neurofilament, phosphorylated tau, and amyloid precursor protein (APP). Recently, we have shown that proteins important for several essential cellular homeostasis networks, such as the autophagy-endosomal system, ER tubulation, and ubiquitin proteasome machinery, are abnormally accumulated in DNs at different time points in three distinguishable layers during Aβ plaque growth [11]. An early autophagy protein ATG9A is found in the 1st layer of DNs at the initial stage of Aβ plaque development. This is followed by the accumulation of ER tubules, as detected by anti-reticulon-3 (RTN3), and clustered mitochondria in the 2nd layer. The 3rd and outer-most layer of DNs contains the late autophagy/endosomal proteins such as RAB7 and LC3.

Lysosomes are major cellular degradative organelles that contain ~ 25 membrane proteins and more than 60 soluble proteins in lumen [22]. The majority of lysosomal luminal proteins are acid hydrolases, which are actively involved in the degradation and recycling of several classes of macromolecules such as proteins, lipids, polysaccharides, and nucleic acids, delivered through autophagy, endocytosis, or other cellular trafficking pathways [22, 23]. Two luminal proteins, lysosomal luminal membrane proteins 1 and 2 (LAMP1 and LAMP2) are routinely used to label endo-lysosomal vesicles and are entrapped in DNs [15, 24]. Since autophagy and endo-lysosomes are integrated cellular homeostasis processes, it is our interest to understand how lysosomes participate in the formation of DNs in AD brains. In this study, we monitored lysosomal accumulation in DNs during amyloid plaque growth by examining brains from three different AD mouse models: APP knock-in (APP^NL-G-F^), 5xFAD and APP/PS1ΔE9 (PA) mice. LAMP1 and the lysosomal activator/lysosomal restricted proteins, saposins (SAPs), were chosen to define the lysosomal organelles. Our results show that cathepsin-deficient, LAMP1^+^- and SAP-C^+^-primary lysosomes were massively accumulated in the 1st layer of DNs during the initial stage of plaque formation. Notably, the intensity of DN-like lysosomal clusters declined during plaque growth, and diminished more in older AD mouse brains, in which LAMP1 was found mainly in the active lysosomes from microglia, specifically the disease-associated microglia (DAM). Similarly, SAP-C^+^-DNs were also diminishing during plaque growth and were nearly absent at the late stage of AD mouse and AD patients’ brains. Our data suggest that continuing intracellular or extracellular Aβ insults cause early accumulation of lysosomes in DNs and sequential impairments in lysosomal structure and functions in neurons, as reflected by the gradual loss of immuno-reactivity of lysosomal proteins in AD mouse brains. The impaired lysosomal systems may viciously facilitate growth of amyloid plaques. Preserving lysosomal functions early is therefore a potential therapeutic strategy for AD intervention.

## Materials and methods

### Mouse strains and AD postmortem brain tissues

APP^NL-G-F^ mice were obtained from the RIKEN Center for Brain Science, Wako, Japan [25]. 5xFAD and Tg-APPsw/PSEN1DE9 (PA) mice were purchased from Jackson Laboratory (stock # 34840 and 004462, respectively). All mice in the study were maintained and used according to protocols approved by the Institutional Animal Care and Use Committee of the University of Connecticut. AD postmortem brain samples were obtained from the NIH NeuroBioBank (Harvard Brain Tissue Resource Center; Human Brain and spinal Fluid Resource Center, Los Angeles; Mount Sinai NBTR Tissue Distribution; University of Maryland Brain and Tissue Bank; University of Miami Brain Endowment Bank).

### Immunohistochemistry and immunofluorescent confocal microscopy

A standard method of immunohistochemistry and immuno-confocal experiments was performed as previously described [26]. Briefly, mouse brains were dissected and fixed with 4% paraformaldehyde fixation for 12 h and immersed in 20% sucrose overnight at 4 °C. The fixed brain tissue was then sectioned in the sagittal plane at a 14 μm thickness using a cryostat after O.C.T. compound embedding. Brain sections were stored at − 20 °C. After three washes with phosphate-buffered saline (PBS), the sections were permeabilized with 0.3% Triton X-100 for 30 min and rinsed in PBS three times to remove detergent. For 3,3′-diaminobenzidine (DAB) staining, 0.3% H2O2 was added with 0.3% Triton X solution. After being rinsed in PBS two times to remove detergent, antigen retrieval was then performed by heating in a microwave in 0.05 M citrate-buffered saline, pH 6.0, for  2-3 min. The sections were then blocked with 5% normal goat serum and incubated with individual primary antibodies: 6E10 (Covance Research Products Inc. Cat# SIG-39330-200, RRID: AB_662804), ATG9A (Abcam Cat# ab108338, RRID:AB_10863880), β-Actin (Sigma Aldrich Cat# A2228, RRID:AB_476697), ATG9A (Abcam Cat# ab108338 RRID:AB_10863880), β-Galactosidase **(**Thermo Fisher Scientific Cat# A-11132, RRID:AB_221539), β-Glucosidase (Santa Cruz Cat# sc-166,407, RRID:AB_2109068), Calnexin (Sigma Aldrich Cat# C4731, RRID:AB476845), Cathepsin B (Santa Cruz Cat# sc-377,299, RRID:AB_10842446), Cathepsin D (Santa Cruz Cat# sc-377,299, RRID:AB_2827539), EEA1(Millipore Cat# 07–1820, RRID:AB_10615480), GFAP (Thermo Fisher Scientific Cat# 13–0300, RRID:AB_2532994), GFAP/SMI22 (Sigma-Aldrich Cat# G3893, RRID:AB_477010), Iba1 (Wako Cat# 019–19,741, RRID:AB_839504), LAMP1(Abcam Cat# ab24170-rabbit, RRID:AB_775978), LAMP1(Abcam Cat# ab25245-rat, RRID:AB_449893), LAMP2 (Abcam Cat# ab25339, RRID:AB_470455), LPL (Abcam Cat# ab93898, RRID:AB_10562464), Neurofilament L (Millipore Cat# AB9568, RRID:AB_11213875), RAB4A (Santa Cruz Cat#sc-517,263, RRID:AB_2177555), RAB4B (Santa Cruz Cat# sc-271,982), RAB5 (Abcam Cat# ab109534, RRID:AB_10865740), RAB6A (Santa Cruz Cat# sc-81,913, RRID:AB_1128894), RAB7 (Abcam Cat# ab137029, RRID:AB_2629474), RAB9A (Santa Cruz Cat# sc-53,145), RAB11(Abcam Cat# ab95375, RRID:AB_10688715), RTN3 monoclonal (recently developed in the Yan lab), PSAP/SAPs (Proteintech Cat# 10801–1-AP, RRID:AB_2172462), Saposin (Santa Cruz cat# sc-100,584, RRID:AB_1128802), SAP-C (Santa Cruz Cat# sc-374,118, RRID:AB_10915437), SMI31 (Covance Research Products Inc. Cat# SMI-31R-100, RRID:AB_10122491), TGN46 (Abcam Cat# ab2809, RRID:AB_2203290), and Ubiquitin (Sigma-Aldrich Cat# U0508, RRID:AB_477599). After overnight incubation at 4 °C, sections were washed with PBS three times and incubated with secondary antibodies conjugated with Alexa Fluor 488 or Alexa Fluor 568 or Alexa Fluor 633 (Molecular Probes) for 2 h at room temperature. For thioflavin-S (Thio-S) staining, sections were washed after the 2nd antibody treatment and 0.01%  Thio-S solution (in PBS) was added and incubated for 20 mins. The sections were finally washed with PBS 3 times and mounted with Vectasheild mounting medium. For DAB staining, sections were subsequently reacted with the specified primary antibody and corresponding biotinylated secondary antibodies (1:200) and developed according to the protocol from the Vector ABC kit (1:400; Vector Laboratories). Images were examined and captured with a Keyence BZ-X810 fluorescence or a Ziess LSM800 confocal microscope.

### Western blotting

Snap-frozen mouse brain cortices were homogenized on ice in RIPA buffer containing complete protease inhibitors (Roche Diagnostics Cat# 06538304001, Mannheim, Germany). The homogenates were rotated for 30 min at 4 °C to ensure extraction of membrane proteins. After centrifugation at 15000 Å ~ g for 120 min, supernatants were collected and protein concentrations were measured with the bicinchoninic acid protein assay reagent (Thermo Scientific, Grand Island, NY, USA). Equal amounts of protein samples were resolved on 4–12% NuPage Bis-Tris gels purchased from Invitrogen. Following incubation with the indicated primary antibody, an appropriate horseradish peroxidase-conjugated secondary antibody was added. Immunoreactivity was detected by chemiluminescence using Super Signal West PICO reagent (Thermo Scientific). Image-J software was used to quantify the mean gray value for a fixed area of each protein band [27]. The original full-blot images can be found in “Additional File_Full gel blots”.

### Three-dimensional electron microscopy (3D EM)

A 10-month-old 5xFAD mouse was anesthetized by injecting pentobarbital interperitoneally and perfused intracardially with ~ 100 ml of 0.1 m sodium cacodylate buffer containing 4% paraformaldehyde and 2.5% glutaraldehyde, pH 7.4. The left and right hippocampus of the extracted brain were fixed overnight with 0.1 m sodium cacodylate buffer containing 4% paraformaldehyde and 2.5% glutaraldehyde. After washing 3 times with 0.1 M sodium cacodylate, the samples were placed in 0.1% tannic acid for 30 min and then washed with sodium cacodylate solution. Next, the samples were sequentially processed by 2 h incubation with 2% osmium tetroxide/potassium ferrocyanide on ice, followed by a 20 min treatment with freshly-prepared 1% thiocarbohydrazide at 60 °C, and then a 1 h incubation with 2% osmium tetroxide solution on a rotor at room temperature. The samples were then placed in new vials, washed 3 times with distilled water, and placed in saturated uranyl acetate solution overnight at 4 °C. The following day, samples were washed 3 times with distilled water and finally stained with lead aspartate solution (prepared by dissolving 0.066 g of lead nitrate in 10 ml 0.03 m aspartic acid, pH 5.5) at 60 °C for 30 min and washed again with distilled water 3 times. The samples were then dehydrated by dipping the samples twice (5 min each) in a gradient series of freshly-prepared solutions of 50, 75, 85, 95, and 100% ethanol, and finally by placing in anhydrous acetone for 10 min at room temperature. The samples were then transferred to freshly-made 50% Epon resin in propylene prepared by mixing 5 ml propylene to 5 ml of 100% Epon resin formulated as 10 ml EMBed-812, 8 ml dodecenyl succinic anhydride, 4 ml methyl-5-norbornene-2,3-dicarboxylic anhydride, and 0.4 ml 2,4,6-tri (dimethylaminomethyl)-phenol. After incubating for 2 h at room temperature in 50% Epon, the samples were transferred to 100% Epon and rotated for 90 min. Finally, the samples were placed into molds and fresh Epon resin was poured and kept at 60 °C for 2 days for polymerization and solidification.

A sample block of 0.5 × 0.5 × 0.5 mm size for each hippocampal tissue was prepared by trimming the resin using a razor blade. Each sample block was mounted on a pin that was set on the stage in a Zeiss Sigma VP scanning electron microscope equipped with a Gatan 3View in-chamber ultramicrotome and a low kilovolt backscattered electron detector. The diamond knife of the microscope was set to make ∼500 sections at a thickness of 70 nm. Images were generated at 2.0–2.25 kV under standard vacuum conditions using an aperture set at 30 μm and captured at 5 nm/pixel resolution (5000× magnification).

### 3D reconstruction of microglia

Registered image sets were analyzed using Reconstruct software [28]. The properties of each image set were entered manually as 70 nm slice thickness and 5 nm pixel size. Areas for amyloid plaques, microglia, and DNs were traced by manual tracing. Each trace with the same name is considered as one object by the software. After tracing each morphology (e.g., amyloid plaque, microglia, DNs, and dense body), a 3D model for each object was generated.

### Cell cultures and Aβ treatment

Mouse neuroblastoma (N2a) cells were cultured in Dulbecco’s Modified Eagles medium (high glucose) and supplemented with 10%(v/v) fetal bovine serum (FBS, GIBCO Cat # 26140–079) and 1% antibiotics (GIBCO Cat # 15240–062) at 37 °C under 5% CO_2_. The primary neurons from cortex and hippocampus of embryonic stage 18 (E18) wild-type mice were cultured separately in neurobasal medium, supplemented with B21 and Glutamex, for 7 days. Human β-amyloid (Aβ)42 peptide (Millipore Sigma Cat# AG968-1MG) was dissolved in 1% NH_4_OH at a concentration of 440 μM, sonicated for 2 min in a cold-water bath, and immediately stored at − 80 °C after aliquoting as stock. The peptide was then dissolved at the desired concentration in cell culture medium. An equal volume of 1% NH_4_OH was included as a control (buffer or mock). The cells were incubated with Aβ42 peptide for 16 h and the cells were then subjected to either immuno-confocal microscopy or Western blotting.

### Cell fractionation/enrichment of lysosomes

Fractionation of cellular organelles from WT and 5xFAD mouse brains were performed using lysosome isolation kit (Millipore Sigma Cat# LYSISO1) according to the modified protocol [29, 30]. Briefly, whole brain tissue (without cerebellum) in extraction buffer (with protease inhibitor) were homogenized with IKA T8 at 8000–9500 rpm for 5 s × 3 times on ice. The homogenate was centrifuge at 1000 x g for 10 min at 4 °C. The supernatant was centrifuged at 5000 x g for 10 min to precipitate mitochondria. The precipitates were washed with extraction buffer and dissolved in minimum volume of buffer. The supernatant was centrifuged at 20,000 x g for 2 h at 4 °C to precipitate lysosomes. The precipitate was washed and dissolved in minimum amount of buffer. After measuring protein concentration for each sample in mitochondria, lysosomes and cytoplasmic fractions, equal amount of protein was subjected to SDS-PAGE and Western blotting.

## Results

### Hydrolase-deficient primary lysosomes were accumulated in the 1st layer of DNs in AD mouse brains

**Fig. 1:**
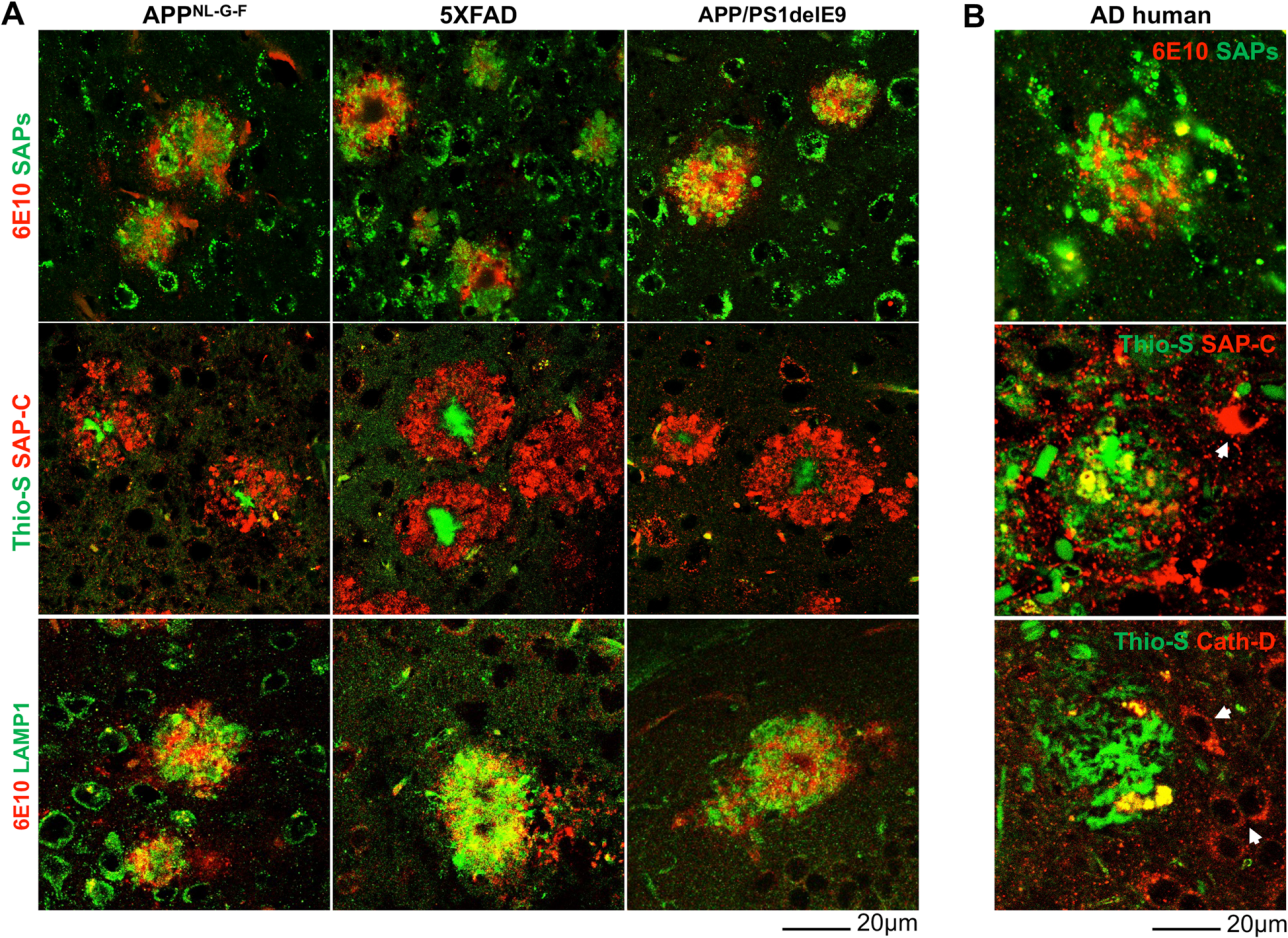
Saposin proteins are enriched in DNs surrounding Aβ plaques in AD brains. **A** Fixed brain sections from 6-month-old APP^NL-G-F^, 4-month-old 5xFAD, and 8-month-old APP-PS19delE9 (PA) mice co-stained with thioflavin-S (Thio-S) or 6E10 and specific antibodies to the lysosomal proteins SAP-C, SAPs, or LAMP1. **B** AD postmortem brain sections were co-stained with  6E10 and SAPs or Thio-S and SAP-C or Cath-D antibodies. Scale bar is shown at the right bottom of each panel

To determine whether DNs contain hydrolase-active or -inactive lysosomes, we stained brain sections from three different AD mouse models, [6-month-old APP knock-in (APP^NL-G-F^), 4-month-old 5xFAD and 8-month-old APP/PS1ΔE9 (PA)], using antibodies specific to several lysosomal proteins including LAMP1 and LAMP2, lysosomal activator protein saposins (SAPs), the SAP precursor (PSAP), lysosomal hydrolases (cathepsin-B, cathepsin-D, β-glucosidase and β-galactosidase), as well as antibodies to the endosomal proteins EEA1, RAB4A/B, RAB5, RAB6A, RAB7, RAB9A, and RAB11. Similar to previous observations in 5xFAD mice [31], LAMP1 antibody could label DNs present in APP^NL-G-F^ and PA mouse brains (Fig. 1A, bottom row). A polyclonal antibody, used to detect PSAP, SAP-A and SAP-B on the western blots but not distinguishing PSAP from SAP-A, SAP-B by immunohistochemical staining, labeled DNs that surrounded amyloid plaques in all three AD mouse models (Fig. 1A, top row, collectively as SAPs). SAP-C, one of the 4 lysosomal activator proteins (SAP-A to -D), is processed from its precursor protein PSAP [32, 33]. A specific SAP-C antibody markedly labeled DNs surrounding amyloid plaques in all examined AD mouse models (Fig. 1A, middle row).

**Fig. 2:**
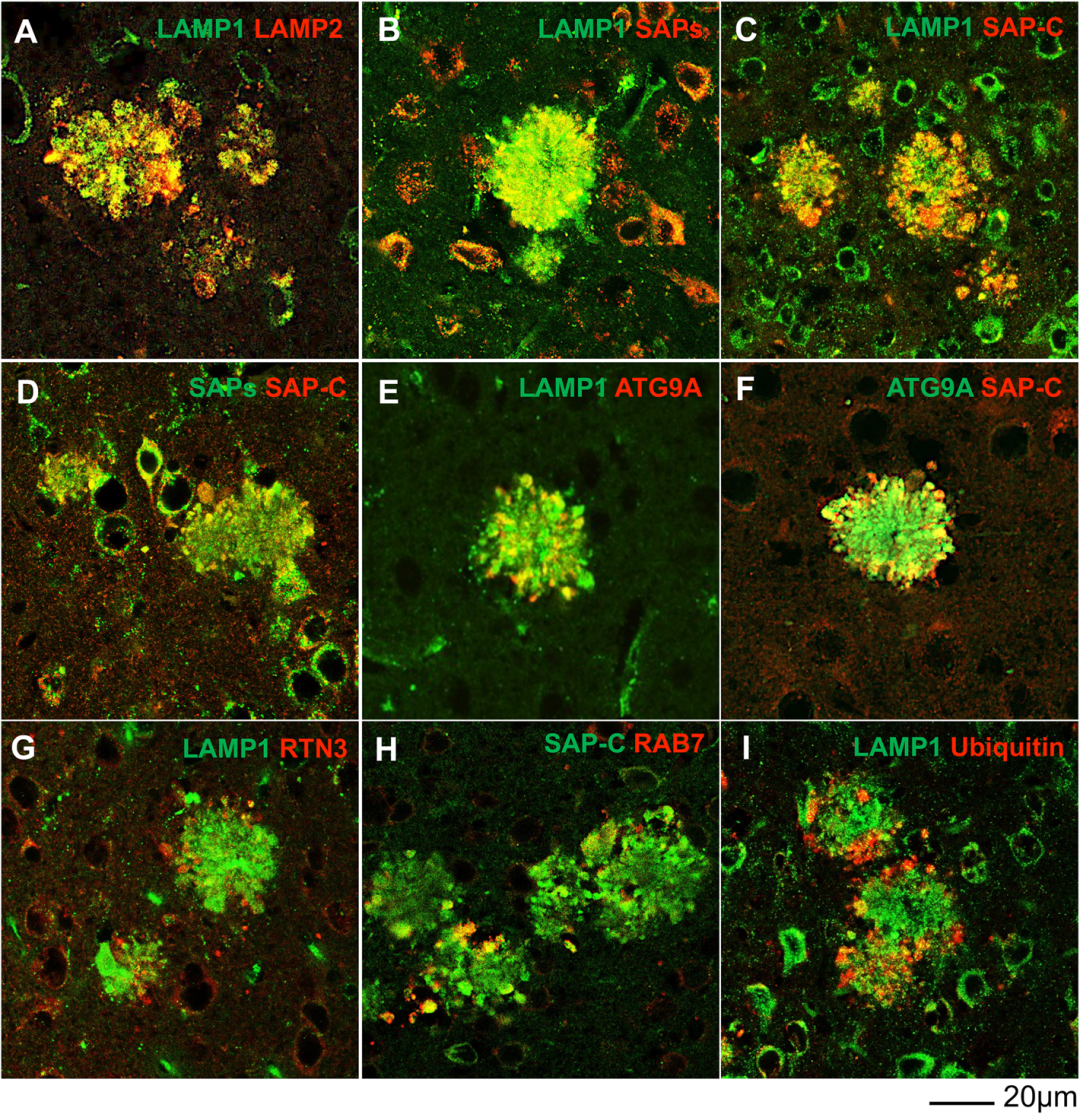
SAP-enriched primary lysosomes are accumulated in the 1st layer of DNs. Fixed brain sections from 6-month-old APP^NL-G-F^ mice were co-labelled with antibodies specific to LAMP1 and LAMP2 **(A)**, LAMP1 and SAPs **(B)**, LAMP1 and SAP-C **(C)**, SAPs and SAP-C **(D)**, LAMP1 and ATG9A **(E)**, ATG9A and SAP-C **(F)**, LAMP1 and RTN3 **(G)**, SAP-C and RAB7 **(H)**, and LAMP1 and ubiquitin **(I)**. LAMP1 and SAP-C were largely co-localized with each other in the 1st layer of DNs, which was labeled by ATG9A, and surrounded by a 2nd layer marker, RTN3, and the 3rd layer markers RAB7 and ubiquitin. Scale bar is shown at the bottom right

We further showed that LAMP1-labeled DNs (LAMP1^+^-DNs) were devoid of cathepsin-B and -D (Supplemental Fig. 1A) or other lysosomal hydrolases such as β-glucosidase and β-galactosidase (data not shown), while both cathepsins B and D were present in the cell body. Among endosomal proteins, we showed that RAB7 antibody labeled DNs in 6-month-old APP^NL-G-F^ mouse brains at a level similar to that in 5xFAD and PA mouse brains [11] (Fig. 2H and supplemental Fig. 1B).

Next, we investigate whether SAP-C, SAPs, and/or LAMP1 could also label DNs in AD human brains. Several AD postmortem brain samples were stained with thioflavin-S (Thio-S) or 6E10 to label amyloid plaques and SAPs, SAP-C, or cathepsin-D antibodies to label DNs (Fig. 1B). SAPs antibody labeled abundance of DNs in surrounding 6E10 labelled plaque area. SAP-C^+^-DNs were also found near Thio-S-stained plaques, although SAP-C antibody also strongly labelled cell bodies in AD postmortem brains (indicated by white arrowheads in Fig. 1B, middle panel). We further conducted triple staining in multiple AD postmortem brain samples with Thio-S, RTN3 and SAP-C antibodies. In an example, RTN3-immunoreactive DNs (RIDNs) were abundantly surrounding amyloid plaques, similar to the previous reports [12, 26], while SAP-C^+^-DNs were relatively less abundant (supplemental Fig. 1C). Cathepsin D antibody did not mark any DNs, but it strongly stained nearby cell body (indicated by white arrowheads in Fig. 1B, bottom panel).

To determine the relationship between LAMP1- and SAPs-labeled DNs, and their localizations in DNs layers, we stained brain sections from 6-month-old APP^NL-G-F^ mice with aforementioned antibodies, and then compared with makers labeling three-layer DNs such as ATG9A, RTN3, RAB7, and ubiquitin [11]. LAMP1 and LAMP2 have significant sequence homology and are known to maintain a dynamic equilibrium among lysosomes and endosomal compartments [34, 35]. LAMP1 and LAMP2 staining were almost completely co-localized in DNs (Fig. 2A). Previous studies showed that SAP-C is localized in ~ 80% of LAMP1^+^ juxtanuclear lysosomes with acidic luminal pH, and in ~ 64% of LAMP1^+^ peripheral lysosomes with relatively less acidity in Hela cells [36], indicating that SAP-C is normally localized in functional lysosomes. LAMP1 was also found to co-localize with SAPs and more prominently with SAP-C (Fig. 2B-D). Interestingly, we did not detect DNs labeled by cathepsin-B or –D (supplemental Fig. 1A). This observation indicated that SAPs^+^- and LAMP1^+^-DNs were enriched with lysosomes that are devoid of cathepsin-B and -D.

Double labeling of LAMP1 or SAP-C with ATG9A, previously-identified to be a 1st layer marker of DNs, showed partial co-localization with a similar pattern of distribution in a comparable radius (Fig. 2E-F), suggesting that LAMP1^+^- and SAP-C^+^-DNs were likely at the 1st layers of DNs in surrounding the Aβ plaque core. This was further confirmed by co-labeling of LAMP1 and RTN3, which is a marker for the 2nd layer of DNs (Fig. 2G), clearly showing a very limited co-localization with LAMP1^+^-DNs surrounded by RTN3-immuno-reactive DNs (RIDNs). In 6-month-old APP^NL-G-F^ mouse brains, the 3rd layer of DNs, as labeled by RAB7 or ubiquitin-specific antibody, clearly localized at the outer periphery of SAP-C^+^- and LAMP1^+^-DNs (Fig. 2H-I). Although SAP-C is also found in late endosomes [33], we observed only a minor co-localization of SAP-C^+^-DNs with RAB7^+^-DNs, suggesting that the accumulation of SAP-C in DNs is mostly likely a processed form in lysosomes. Ubiquitin-labeled DNs were rarely co-localized with LAMP1^+^-DNs, and were clearly located at the outer circle (Fig. 2I). Our results indicate that the 1st layer of DNs in AD mouse brains mainly accumulate ATG9A and lysosomes, likely primary lysosomes, which contain SAPs but no hydrolase such as cathepsin-B or -D.

### Reduced accumulation of lysosomes in DNs during amyloid plaque growth

**Fig. 3:**
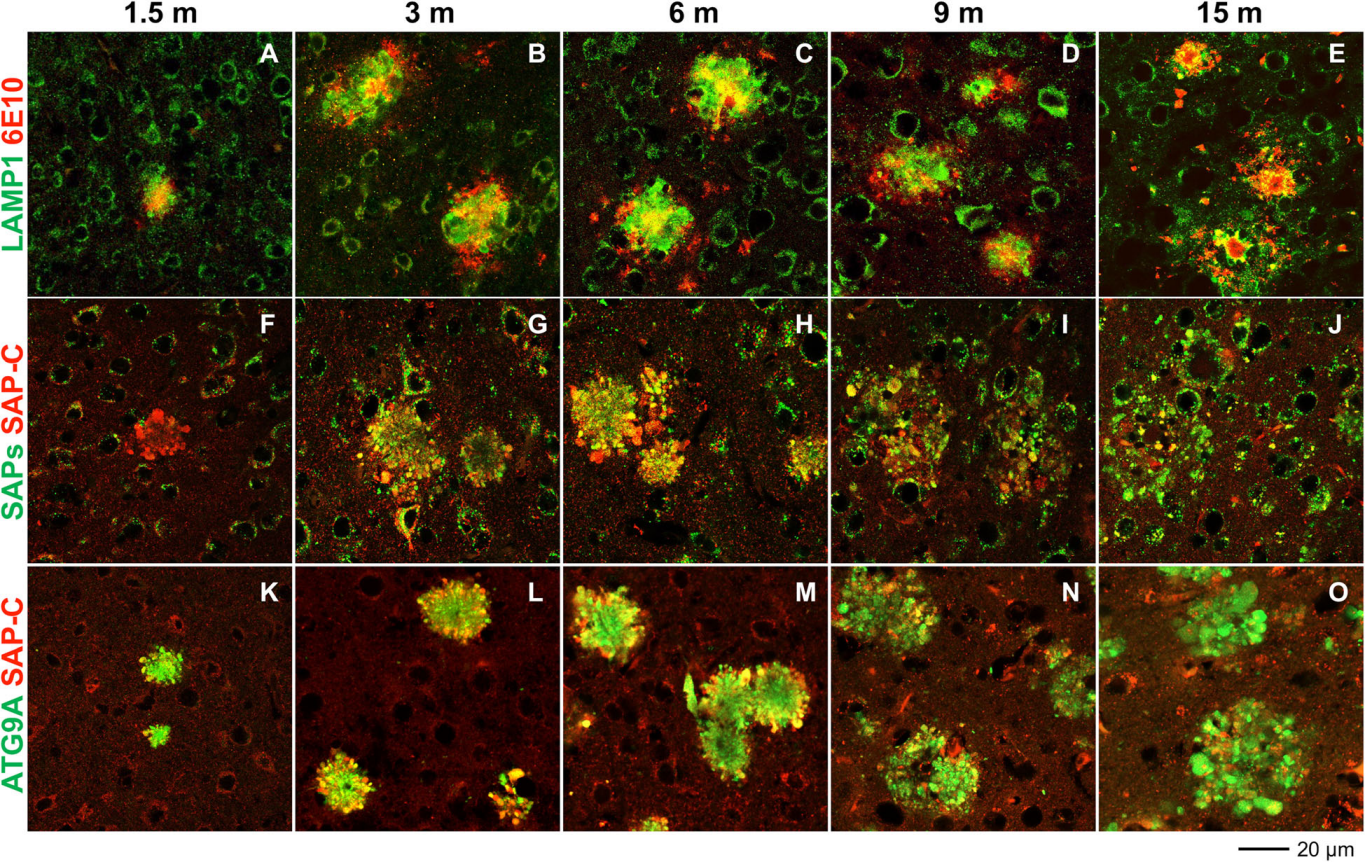
Differential association of lysosomal proteins in DNs during plaque growth in APP^NL-G-F^ mouse brains. Fixed brain sections from 1.5- **(A, F** and **K)**, 3- **(B, G** and **L)**, 6- **(C, H** and **M)**, 9- **(D, I** and **N)** and 15- **(E, J** and **O)** month (m)-old APP^NL-G-F^ mice were double-labeled with antibodies against ß-amyloid plaque (6E10) and LAMP1 **(A-E)**, SAP-C and PSAP **(F-J),** and SAP-C and ATG9A **(K-O)**. Both LAMP1^+^ and SAP-C^+^ DNs appeared at early stages of plaque formation, when ATG9A also labelled DNs. The intensities of LAMP1- and SAP-C-labeled DNs were gradually reduced from 6 m to 15 m. At 15 m, residual LAMP1 staining was apparent and very little SAP-C^+^-DNs existed at this age. ATG9A-labeled DNs were abundant at 15 m

Our earlier immuno-confocal and electron microscopy studies have shown that dysfunction in pre-autophagosome formation, ER tubulation, mitochondrial dynamics, autophagy degradation, and ubiquitin proteasome systems occurs at different time points during amyloid plaque growth in 5xFAD and PA mouse brains, resulting in sequential accumulation of proteins, such as ATG9A, RTN3, RAB7, and ubiquitin, in three layers of DNs in surrounding amyloid plaques [11]. In this study, we monitored the temporal accumulation of LAMP1- and SAPs-positive lysosomal vesicles during different stages of plaque progression, such as the Aβ plaque-forming stage, the plaque pre-stabilization stage (i.e., pre-saturation stage of plaque formation), and the post-plaque saturation stage. Cortical Aβ deposition in homozygous APP^NL-G-F^ starts at ~ 2 months of age and the plaque deposition saturates at ~ 7 months; mice exhibit memory impairments by the age of 6 months [25]. In this mouse model, we found that 6E10 antibody detected very few tiny plaques as early as post-natal day 46 (~ 1.5 months) in the cortical area of female APP^NL-G-F^ homozygous mice. Hence, we used female homozygous APP^NL-G-F^ mice for most of our subsequent studies and determined 1.5 months as the initial plaque-forming stage. We viewed 6 months old APP^NL-G-F^ mice as the pre-plaque saturation stage, and designated 9 months and 15 months ages as the post-plaque stabilization stages. We stained the brain sections from these mice to label amyloid plaques (by 6E10 antibody) and lysosome-containing DNs (LAMP1, SAP-C, SAPS, cathepsin B, and cathepsin D), and compared the lysosomes-labeled DNs with ATG9A- and RTN3-labeled DNs (Fig. 3A-O, supplemental Fig. 2A-B). At 1.5 months, LAMP1-, SAP-C-, and ATG9A-specific antibodies were able to detect DNs even when a tiny Aβ plaque appeared in frontal cortex (Fig. 3A, F, and K). Differentially, SAPs antibody did not visibly label DNs at 1.5 months, but it appeared at 3 months when RTN3^+^-DNs began to appear (supplemental Fig. 2A). We noted that LAMP1^+^-, SAP-C^+^-, and SAPs^+^-DNs were increased until 6 months but became reduced at 9 months and further declined at 15 months (Fig. 3A-J, supplemental Fig. 2A). A residual of LAMP1^+^- and SAPs^+^-DNs were detectable at 15 months, but noticeably, the staining was predominantly localized at the center of the plaque (Fig. 3E and supplemental Fig. 2A). At this time point, SAP-C^+^-DNs were largely diminished from many plaques (Fig. 3J). Double-labeling of SAP-C and ATG9A showed a selective reduction of SAP-C^+^-DNs, while ATG9A^+^-DNs were actually growing larger in older (9 months and 15 months) APP^NL-G-F^ mouse brains (Fig. 3N-O). Similarly, 5xFAD and PA mouse brains also showed a selective reduction in intensity of LAMP1^+^-DNs in older mouse brains compared to ATG9A^+^- and RTN3^+^-DNs, while LAMP1^+^ signals were shifted to the central plaque localization (supplemental Fig. 2B). We noticed that cathepsin B and cathepsin D antibodies labeled lysosome-like organelles at 15 months and were completely co-localized with LAMP1^+^ signals at the central plaque area (supplemental Fig. 2C).

Although a neuritic plaque size was visibly growing in an age-dependent manner, the intensity of DNs varied in different cases. We noted that the image intensity of LAMP1^+^-DNs, ATG9A^+^-DNs, or SAP-C^+^-DNs in different age groups was clearly differential: the intensity of ATG9A^+^-DNs was stronger with aging while the intensity of LAMP1^+^-DNs or SAP-C^+^-DNs was comparably weaker with aging (i.e, comparing the size of SAP-C^+^-DNs in 3F vs 3 J). This reduction of DNs enriched with lysosomal proteins was likely due to the loss of detectable epitopes or degradation of these proteins.

**Fig. 4:**
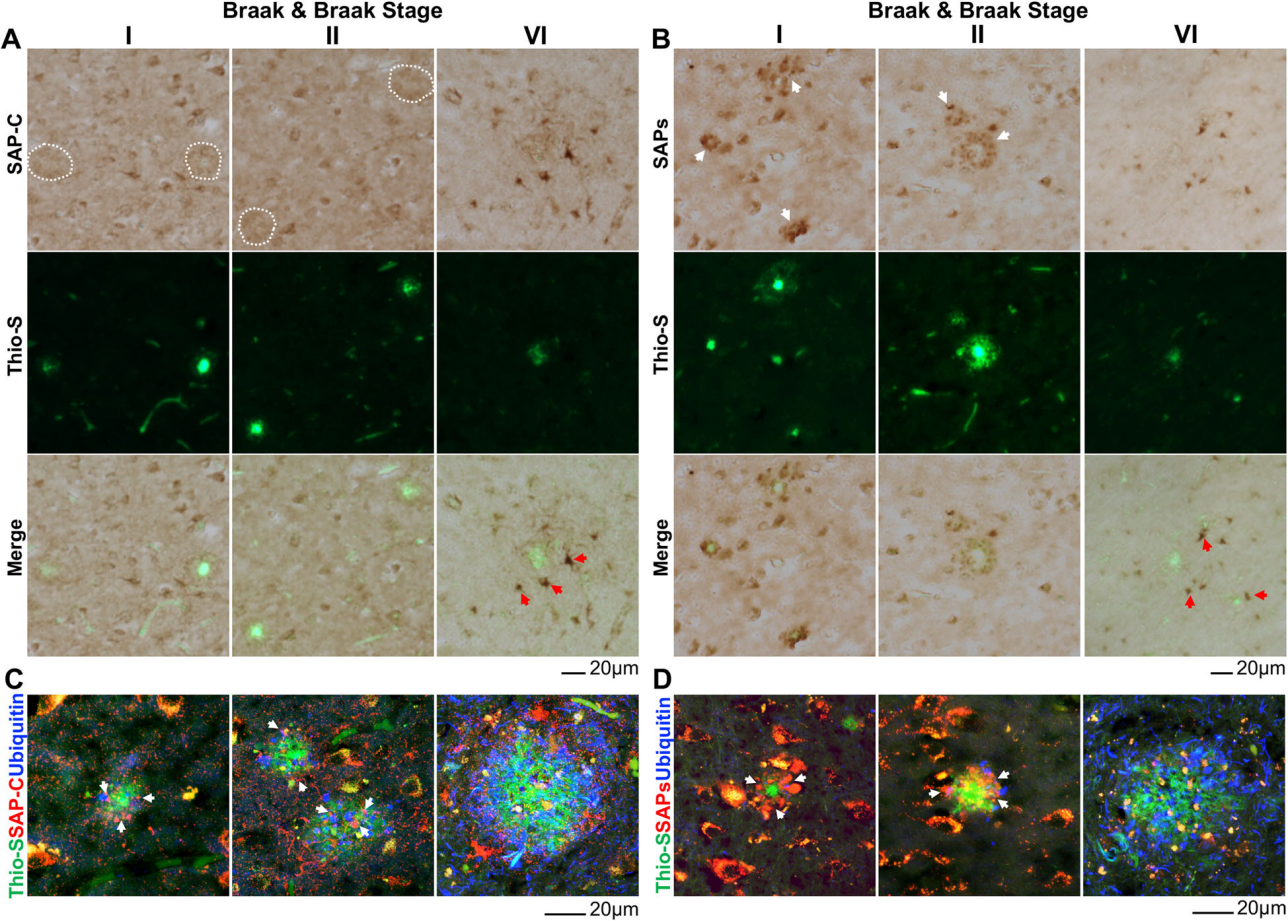
Labeling DNs by SAP-C and SAPs at early stage of AD progression in human postmortem brain. **A**-**B** Fixed brain section from hippocampus area of Braak & Braak stages I, II and VI AD patients were DAB stained with either  SAP-C **(A)** or SAPs **(B)** antibodies. Amyloid plaques were labeled by thioflavin (Thio-S) staining. SAP-C^+^-DNs were marked by dotted circles while SAPs^+^-DNs were indicated by white arrowheads. **C**-**D** AD brain samples were also examined by immunofluorescent staining with SAP-C **(C)** or SAPs **(D)** to mark these DNs. Ubiquitin antibody marked outer layer 3 DNs while Thio-S was used to mark amyloid plaques. Scale bar shown at the bottom of each panel. SAPs antibodies labeled DNs in Braak & Braak stages I and II samples, but much fewer in Braak & Braak stage VI samples

To examine whether the formation of SAPs^+^-DNs follow similar pattern during disease progression in AD human brains, we performed a combination of DAB and fluorescence staining using AD postmortem brain samples at different Braak & Braak (B & B) stages (supplemental Table 1). First, DNs were marked by DAB staining with SAP-C or SAPs antibody to detect SAP-C^+^-DNs or PSAP^+^-DNs. Amyloid plaques were subsequently labelled by Thio-S staining (Fig. 4A and B). Although, Braak staging is based on tau pathology [37], we were able to correlate amyloid plaque loads with progressive pathological states in our hippocampal samples. SAP-C^+^-DNs surrounding Thio-S^+^ plaques were traceable in early stage of disease, *i., e.*, B & B stage I and II, (indicated by white dotted circle in Fig. 4A) and those DNs were clearly visible when labeled by SAPs antibody (indicated by white arrowhead in Fig. 4B). A triple staining with SAP-C or SAPs, ubiquitin and Thio-S also showed SAP-C^+^-DNs and SAPs^+^-DNs in B & B stage I and II samples (indicated by white arrowhead in Fig. 4C-D). Ubiquitin antibody labeled DNs at outer periphery of SAP-C^+^- and SAPs^+^-DNs surrounding Thio-S^+^ core (Fig. 4C-D). On the contrary, SAP-C or SAPs antibody did not mark DNs in Thio-S^+^ plaque vicinity area in B & B stage VI sample while it strongly stained nearby cells (indicated by red arrowhead in Fig. 4A-B). At this stage, ubiquitin marked DNs were abundant (Fig. 4C-D). These results are consistent with data in our mouse models, demonstrating that SAPs-enriched lysosomes are abnormally accumulated in the first layer of DNs at early stage, but they diminished later during AD pathogenic progression in human brains.

### Origin of LAMP1^+^ and SAP-C^+^-lysosomes surrounding Aβ plaques

**Fig. 5:**
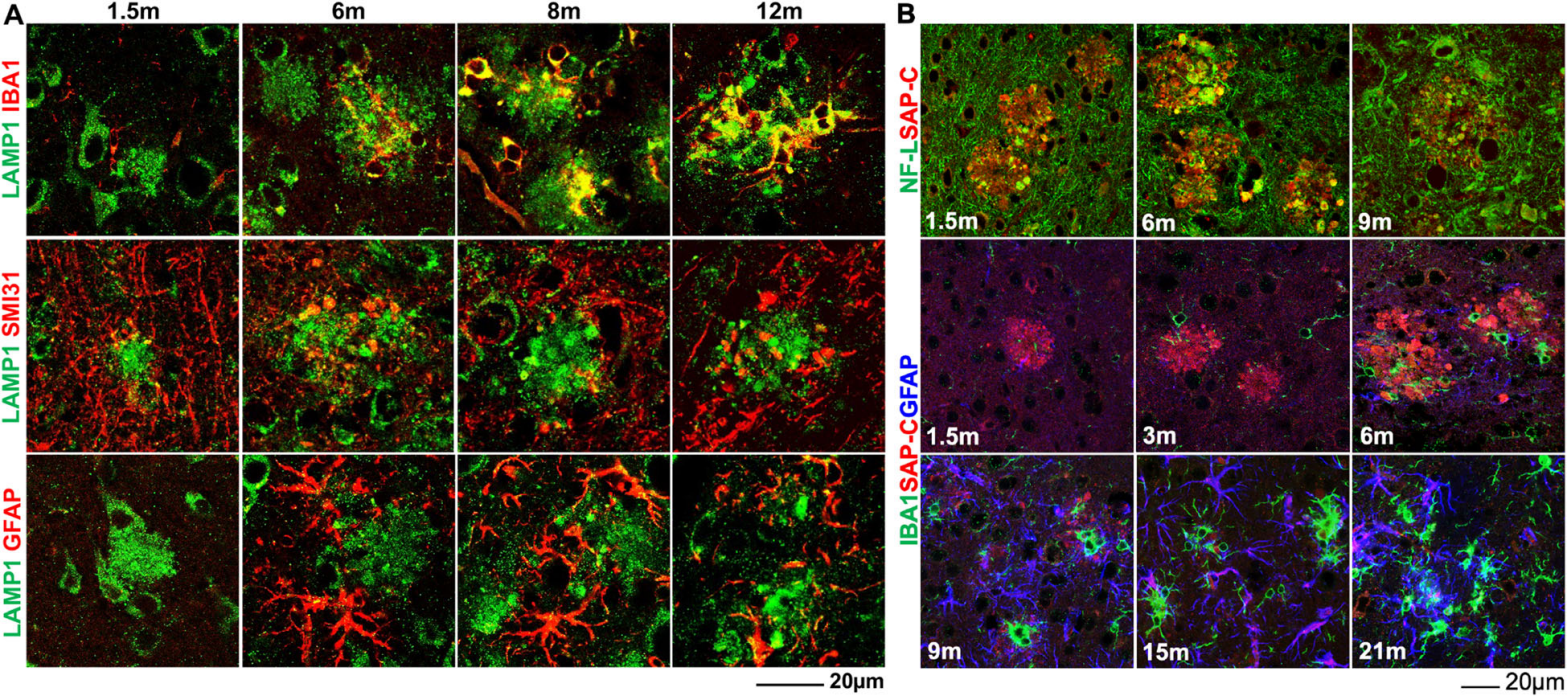
Neuronal and glial localization of LAMP1- and SAP-C-labelled lysosomes surrounding the Aβ plaque area. **A** Fixed brain samples from 1.5-, 6-, 8-, and 12-month (m)-old APP^NL-G-F^ mice were co-stained with LAMP1 and IBA1, SAMI31, or GFAP antibodies. At 1.5 m and 6 m, LAMP1^+^-DNs were co-labeled with SMI31, but in older mouse brains (12 m), LAMP1 largely co-localized with IBA1. **B**  Fixed brain sections from 1.5-, 6-, and 9-m-old APP^NL-G-F^ mice were co-stained with SAP-C and neurofilament L (NFL) antibodies, and brain sections from 1.5-, 3-, 6-, 9-, 15 and 21-m-old APP^NL-G-F^ mice were triple-stained with SAP-C, IBA1, and GFAP. SAP-C was largely co-localized with NFL at swelled dystrophic axons, but it did not co-label with IBA1, even at older ages. However, strong labeling of SAP-C in astrocytes in the plaque vicinity, as labeled by GFAP, was evident in older mouse brains

We showed that LAMP^+^-DNs were rapidly increased during early plaque growth stages and then were gradually reduced after stabilization of plaque formation in APP^NL-G-F^, 5xFAD, and PA mouse brains. We also observed that residual LAMP1 staining completely co-localized with cathepsin-labeled lysosome-like organelles in older APP^NL-G-F^ mouse brains (supplemental Fig. 2C). To resolve this ambiguity and to determine the origin of LAMP1^+^-DNs or immunity in surrounding Aβ plaques during plaque growth, we co- and triple-stained brain sections from 1.5-, 3−/6-, 8−/9-, and 12−/15−/21-month-old APP^NL-G-F^ mice using antibodies to label microglia (IBA1), neurofilaments (SMI31/NF-L) or astrocytes (GFAP) in addition to LAMP1 or SAP-C antibody (Fig. 5A-B). Co-labeling 1.5-month-old APP^NL-G-F^ mouse brain sections with IBA1 and LAMP1 or SAP-C antibodies showed the appearance of a few microglial cell bodies near plaques (Fig. 5A-B). More microglia were found near plaques at 3 months, with microglial soma in contact with SAP-C^+^-DNs (Fig. 5B). The number of IBA1^+^ microglia was gradually increased during plaque growth and clustered near plaques, i.e., at the age of 9 months and 12 months (Fig. 5A). At 1.5 months and 6 months, LAMP1^+^ DNs were largely co-localized with a neurofilament marker, SAMI31, at swelled neurites or bulb-like structures (Fig. 5A). Co-labeling of SAP-C with neurofilament L (NF-L) also showed a co-localization of SAP-C at the neuronal bulb-like bouton during these stages (Fig. 5B), suggesting a predominant neuronal origin of lysosomes in DNs.

In magnified images, we also noticed that the morphology of SMI31-labelled neurofilaments was intact and that many LAMP1-labelled lysosomal puncta were visible in axons (as indicated by white arrowheads in Supplemental Fig. 3), reflecting functional lysosomes trafficking through axons [38]. When plaque formation proceeded toward saturation (6 months), such LAMP1 localization in intact neurofilaments was reduced, and SMI31-labelled neurofilaments surrounding plaques were mostly destroyed at the post-plaque saturation stage (9 months); lysosomal puncta in neurofilaments were rarely noticeable at this late stage (Fig. 5A, supplemental Fig. 3).

On the other hand, co-localization between LAMP1^+^ and IBA1^+^ immunity in surrounding plaque areas was gradually increased, and it was more evident at 12 months, as LAMP1 staining was mostly co-labeled with IBA1 staining near central plaque areas (Fig. 5A). A similar pattern of LAMP1 and IBA1 staining was also seen in 5xFAD and PA mouse brains, where LAMP1 staining was largely co-localized with IBA1 staining in surrounding amyloid plaques in older mouse brains (supplemental Fig. 4A-B). Triple staining of IBA1, ATG9A, and LAMP1 in brain sections from older mice from all three AD models showed selective localization of LAMP1 in microglia, while ATG9A was barely co-localized with IBA1 (supplemental Fig. 4C). IBA1, LAMP1, and cathepsin-D triple-labeling of 15-month-old APP^NL-G-F^ mouse brain sections showed clear localization of LAMP1 and cathepsin in plaque-associated microglia (supplemental Fig. 4D). All of these results indicate that LAMP1 staining in older AD mouse brains mainly labels the cathepsin-positive active lysosomes in microglia that are near amyloid plaques.

The appearance of reactive astrocytes at the plaque was a comparably later event than the arrival of microglia (Fig. 5B). Astrocytes with reactive morphology were clearly visible at 6 months (Fig. 5A-B). However, co-localization of LAMP1^+^-DNs with GFAP was sparse, except for a few in the cell body, which were likely related to active lysosomes in astrocytes (Fig. 5A). SAP-C was scarcely co-localized with IBA1, even at 9 months when residual SAP-C^+^-DNs were still detectable. Interestingly, SAP-C was localized in many astrocytes in older APP^NL-G-F^ mouse brains, when SAP-C^+^ DNs were diminishing at 15- and 21-month-time points (Fig. 5B).

**Fig. 6:**
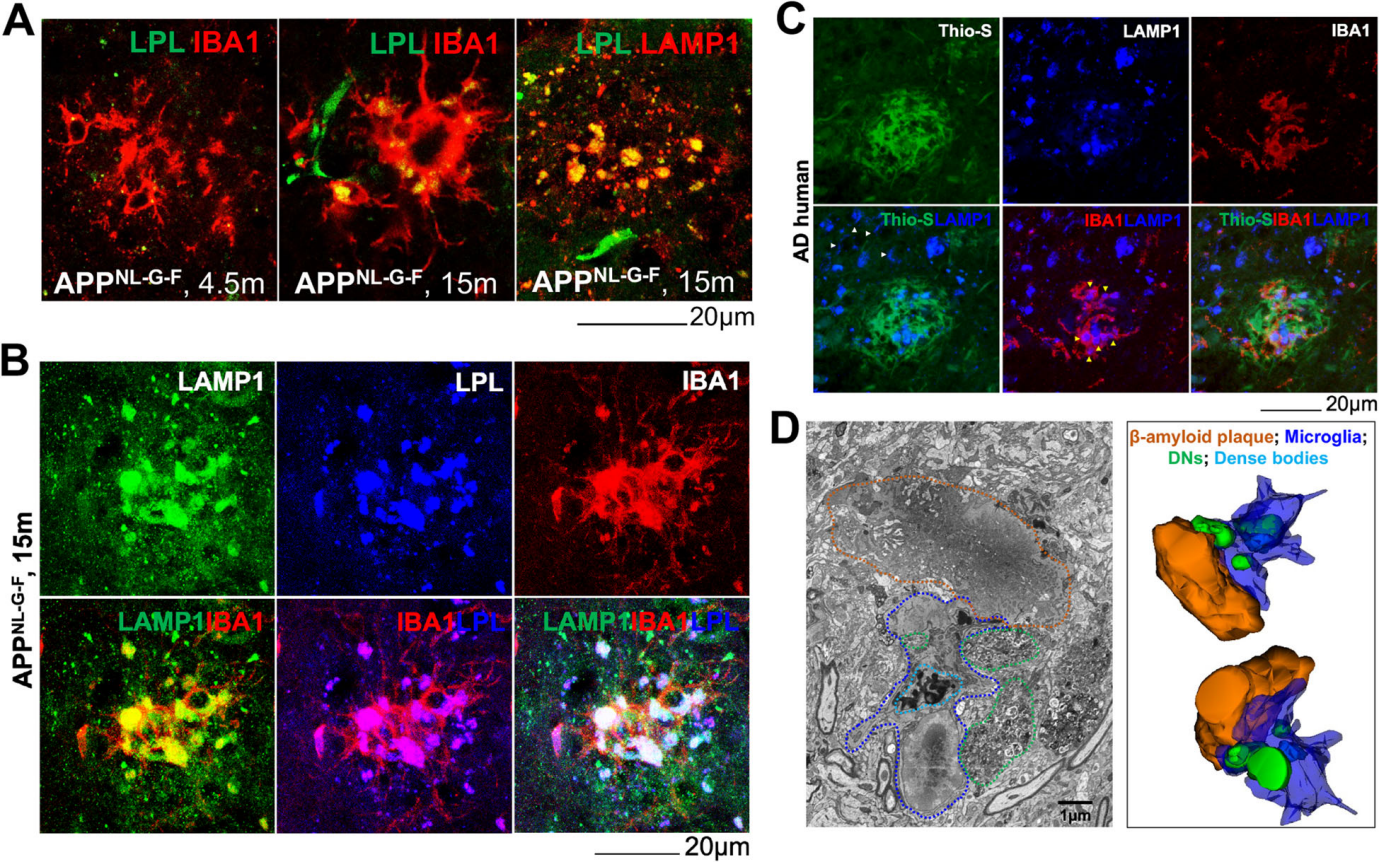
LAMP1-labeled phagocytic DAM surrounding plaque in AD brains. **A** Brain sections from 4- and 15-month (m)-old APP^NL-G-F^ mice were co-stained with IBA1 and LPL or sections from 15-m-old APP^NL-G-F^ mice were co-stained with LAMP1 and LPL. **B** Fixed brain sections from 15-m-old APP^NL-G-F^ mice were triple-stained with LAMP1, IBA1, and LPL. **C** AD postmortem brain sections were triple-stained with Thio-S, IBA1, and LAMP1. **D** Fixed and processed blocks of brains from 10-m-old 5xFAD mice were subjected to 3D electron microscopy (3D EM). Microglia (blue dashed line), dense bodies (turquoise dashed line), and DNs (green dashed line) in close proximity to an amyloid plaque (orange dashed line) are shown on a single micrograph from 3D-EM image stacks. Filopodia-like morphology in microglia engulfing DNs is evident in the image. Amyloid plaque (orange), microglia (blue), dense bodies inside microglia (turquoise), and DNs (green) were tracked through a stack of ~ 100 images and a 3D structure of the whole plaque was reconstructed using Reconstruct software

We also asked whether LAMP1-labeled microglia at the plaques in older APP^NL-G-F^ mouse brains were disease-associated microglia (DAM), a term described previously [39]. Double-labelling of 4-month-old APP^NL-G-F^ mouse brain sections with IBA1 and a phagocytic DAM marker LPL showed tiny droplet-shaped staining of LPL in IBA^+^ microglia (Fig. 6A). The size of LPL-labelled droplets in microglia was enlarged at the age of 15 months. Co-staining of LPL and LAMP1 in 15 months sections showed complete co-localization of those LPL droplets with LAMP1 staining. Triple staining of 15-month-old APP^NL-G-F^ mouse brain sections with LAMP1, LPL, and IBA1 confirmed the major localization of LAMP1 and LPL in microglia (Fig. 6B). LAMP1 staining in AD postmortem brain was also localized in DAM, as demonstrated by triple-staining with Thio-S, LAMP1, and IBA1 (Fig. 6C). LAMP1 immunoreactivity was largely localized in microphages at the plaque (indicated by yellow arrowheads in Fig. 6C), with a similar staining pattern observed in 15-month-old APP^NL-G-F^ mouse brain (Fig. 6B-C). At a distal region from the plaque, LAMP1 stained cell bodies, probably neuronal soma (indicated by white arrowheads in Fig. 6C). Hence, our data indicate that strong LAMP1 staining in old AD mouse and late-stage AD human brains is mostly associated with DAM.

We further utilized 3D-electron microscopy (3D-EM) to investigate whether DAMs mediate phagocytosis of DNs. A typical microglial morphology (indicated by the blue dashed line) with a nucleus and extended processes in the plaque vicinity area (indicated by the orange dashed line) was recognizable in a typical 2D electron-micrograph, selected from 3D serial image stacks obtained from 10-month-old 5xFAD mouse brains (Fig. 6D). A dense inclusion body (indicated by the turquoise dashed lines) was seen within microglia, likely related to an aggregated material-containing large lysosome/phagosome in a DAM. Few microglial sprouts were found to extend toward DNs (indicated by the green dashed line), likely forming filopodia-like structures, which is a characteristic engulfing pattern of large cellular particles by macrophages [40]. We also noticed a trace of DN morphology (indicated by the green dashed line) within microglia on selected 2D EM pictures (Fig. 6D).

To determine the interaction between microglia and DNs, we analyzed more than 100 3D-EM images and reconstructed a 3D model using reconstruct software [28]. The reconstructed-3D structure showed that microglial processes were extended to the surface of the amyloid core with some sprouts wrapping around DNs (Fig. 6D, right panel). Our data therefore suggest that LAMP1^+^ DAMs likely medicate phagocytosis of DNs.

### Dynamic changes in lysosomal protein composition and levels during plaque growth

**Fig. 7:**
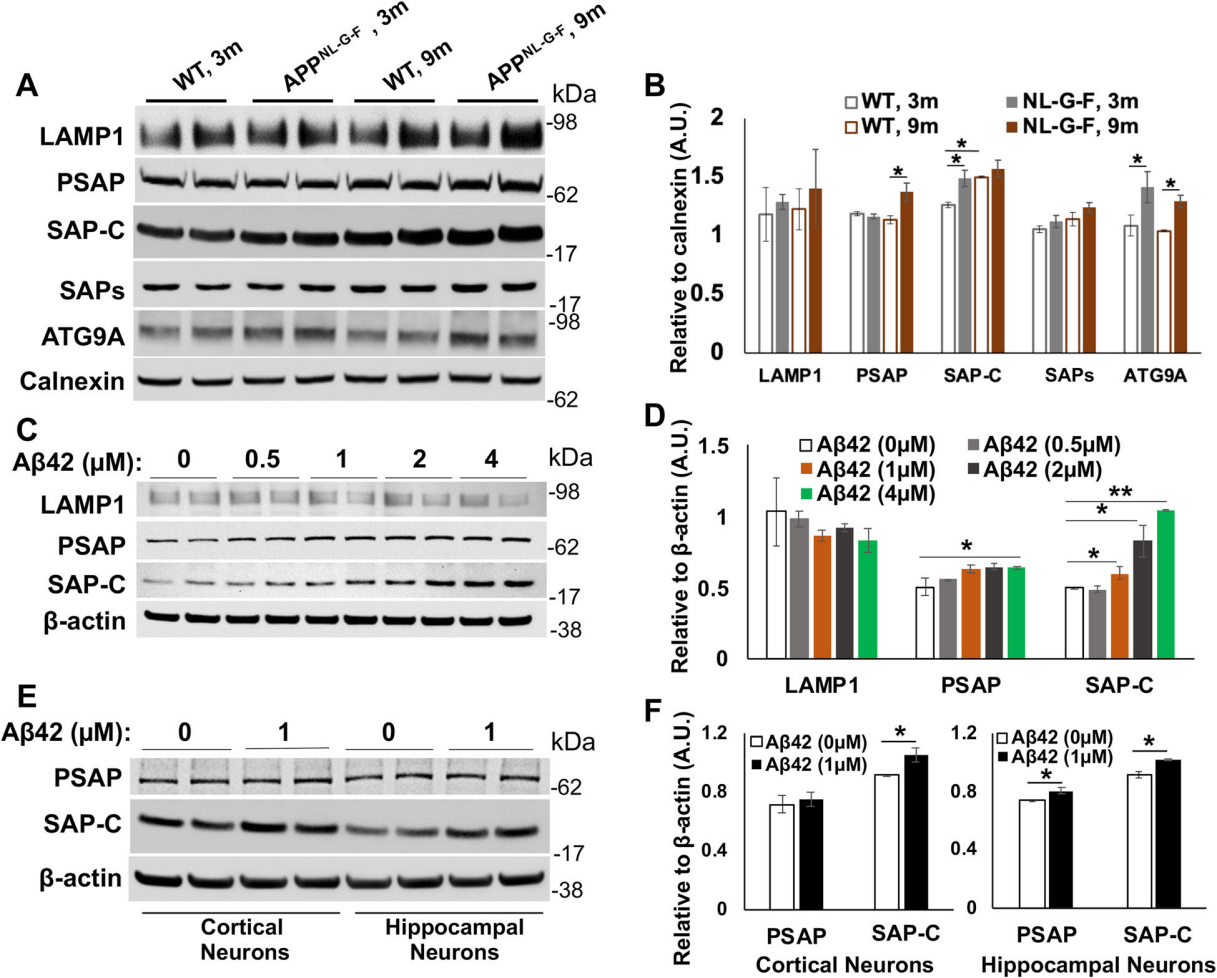
Alteration in protein levels of DN-forming proteins in APP^NL-G-F^ mouse brains and in vitro Aβ42 treated cells/neurons. **A** Protein lysates from the cortex of 3-month (3 m) and 9-month (9 m) old WT and APP^NL-G-F^ mice were prepared for Western blot analysis with antibodies to LAMP1, PSAP, SAP-C, SAPs, ATG9A and calnexin. **B** Quantification for each protein band was conducted using Fiji software and the average of each age group was normalized to the calnexin level. **C** Lysates prepared from N2a cells treated with vehicle only (0) or Aβ42 (0.5 to 4 μM) for 16 h (h) were subjected to Western blot analysis. **D** Band intensity for each protein was measured and normalized to the intensity of β-actin. **E**, **F** Primary neurons, cultured form the cortical and hippocampal regions, were also treated with vehicle or 1 μM Aβ42 for 16 h (h), and protein lysates were examined by Western blot analysis **(E)** and similar quantification **(F)**. *N* = 3 independent experiments in all western analyses (* *P* < 0.05, ** *P* < 0.01, student t-test)

With the shift of staining patterns labeled by lysosomal proteins in DNs, we examined protein levels of various ages of female APP^NL-G-F^ homozygous mice, 5xFAD mice and their WT littermates. Cortical protein lysates were used for Western blots with antibodies to LAMP1, PSAP, SAP-C, SAPs, and ATG9A. Among these proteins, ATG9A levels were increased in both 3- and 9-month-old APP^NL-G-F^ mouse brains compared to their WT controls (Fig. 7A-B), consistent with increased accumulation of ATG9A in DNs in AD brains. We also noticed a significant increase in PSAP protein levels in APP^NL-G-F^ mouse brains compared to WT controls, but this change was only evident in older mouse brains (Fig. 7A-B). Protein levels of SAP-C in 3-month-old APP^NL-G-F^ mouse brains were significantly increased compared to WT controls, but this increase was less significant at 9 months old (Fig. 7A-B), likely related to age-dependent increase of SAP-C in general. LAMP1 and  SAPs (mainly SAP-A/B) protein levels were less obviously altered among the different groups (Fig. 7A-B).

During plaque growth, LAMP1, SAPs, SAP-C, and ATG9A protein levels appeared to be significantly increased in both APP^NL-G-F^ (Supplemental Fig. 5A-B) and 5xFAD mouse brains (Supplemental Fig. 5C-D). Protein levels of PSAP were relatively stable during aging in both AD mouse models. Unlike ATG9A, LAMP1 and SAP-C were not richly accumulated in DNs in older 5xFAD and APP^NL-G-F^ mouse brains, and their protein levels were actually increased rather than decreased or degraded. Hence, selective decline of lysosomal proteins in DNs of aged plaques was not due to a reduction in these protein levels.

Next, we asked whether an increase of saposin protein levels in APP^NL-G-F^ mouse cortices compared to their WT controls and in AD mouse brains during plaque growth could be the consequence of Aβ accumulation in neurons. To this end, we treated mouse neuroblastoma-2a (N2a) cells with different concentrations of Aβ42 for 16 h (h) and measured the protein levels of LAMP1, PSAP, and SAP-C (Fig. 7C-D). While no significant changes were observed in LAMP1 protein levels in Aβ42 (0.5 to 4 μM)-treated cells, a dose-dependent increase in SAP-C protein levels was evident, with a significant increment starting at 1 μM concentration of Aβ42 (Fig. 7C-D). We similarly treated primary cortical and hippocampal neurons (7 days old), cultured from WT mouse embryos day 18 (E18), and observed a significant increase in SAP-C protein levels at 1 μM of Aβ42 treatment compared to vehicle treatment (0 μM of Aβ42 in Fig. 7E-F). These results indicate that increased toxic Aβ treatment could significantly enhance SAP-C protein levels.

### Enhanced lysosomal SAPs induce the lysosomal impairment in AD mouse brain

**Fig. 8:**
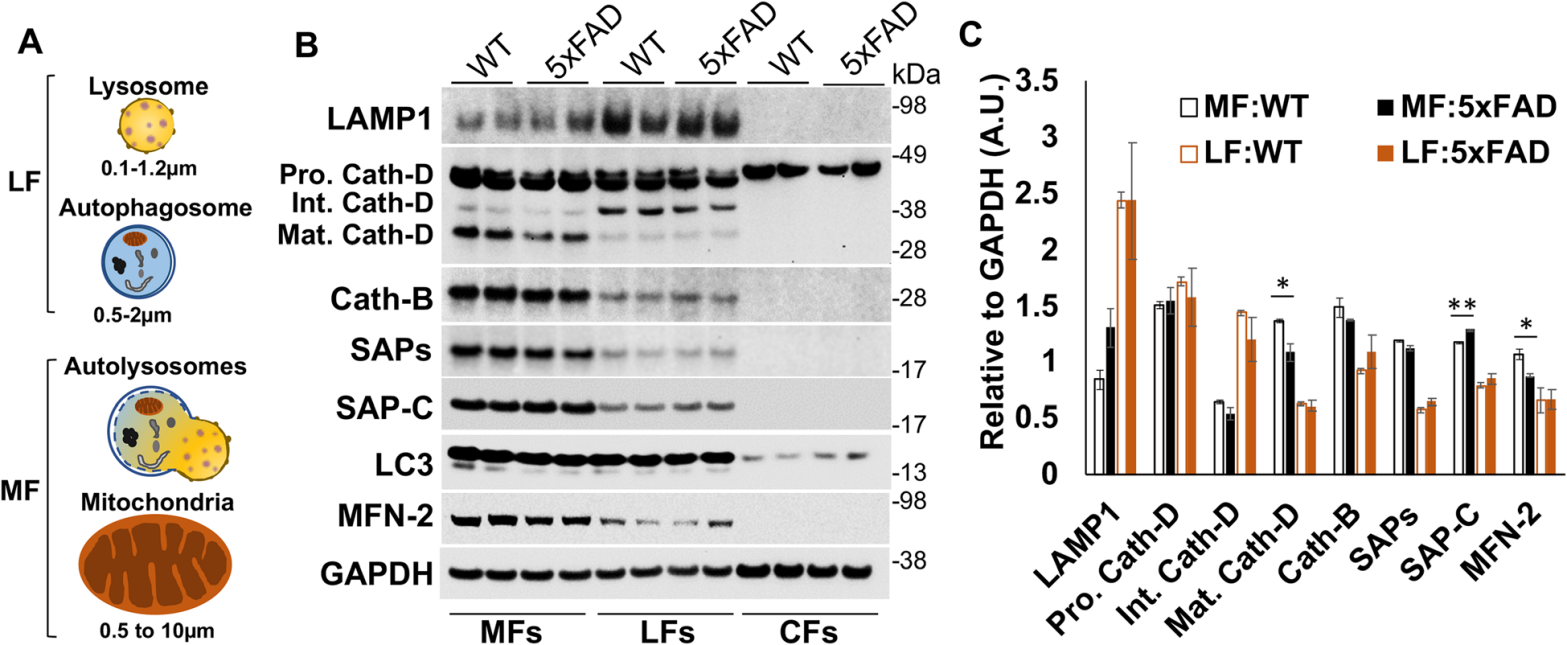
Alteration of SAP-C and cathepsin-D protein levels in lysosomes or autolysosomes in AD mouse brains. **A** A schematic diagram showing the variable size of lysosomes, autophagosome and mitochondria enriched in the fractionation experiment. Fusion of a lysosomes and autophagosome during autophagy-lysosomal degradation produce larger autolysosome organelles. Variable sized organelles were separated in mitochondria - and lysosomes-enriched fractions (MFs or LFs) by sequential centrifugation. The final supernatant is designated as cytosolic fractions (CFs). **B** Proteins in MFs, LFs and CFs were analyzed on the Western blot with the indicated antibodies. **C** Band intensity for each protein was measured and normalized by the GAPDH level (*N* = 3 independent experiments, * *P* < 0.05, ** *P* < 0.01, student t-test). SAP-C protein level was increased, but mature cathepsin-D level was reduced in autolysosomes in 5xFAD mouse brains

Our western results showed that SAP-C protein levels were significantly increased in APP^NL-G-F^ mouse brains compared to WT beginning at younger ages, and in Aβ42 treated N2a cells or primary cultured neurons compared to non-treated groups. As demonstrated previously, an increased SAP-C in lysosomes likely activates lysosomal membrane permeabilization (LMP) and induces LMP-mediated lysosomal damages [41–43]. To determine whether an increment in lysosomal SAP-C levels in AD mouse brains affects lysosomal integrity, we performed cell fractionation assays using 2-month-old WT and 5xFAD mouse brains (Fig. 8). The cellular organelles were separated into mitochondrial-enriched fractionations (MFs) and lysosomes-enriched fractions (LFs) by gradient centrifugation as previously described [29, 30] (Fig. 8A). LAMP1 was relatively enriched in LFs, which contain mostly lysosomal vesicles and autophagosomes, while an increase of mitofusion-2 (MFN2) in MFs indicated mitochondrial enrichment in this fraction. Noticeably, MFs contained fused vesicles, which are related to the larger size of LC3^+^ and LAMP1^+^ autolysosomes formed by fusion of autophagosomes and lysosomes during autophagy-lysosomal degradation processes [44] (Fig. 8A-B). Seemingly, LFs likely contained smaller sizes of mitochondria, as reflected by the appearance of mitochondrial protein MFN2.

With this relative enrichment, we observed that full length cathepsin-D, which appeared in double bands at the size of ~ 45/47 kDa as the non-glycosylated and mono-glycosylated pro forms [45], was almost equally enriched in MFs and LFs (Fig. 8B). The processed mature cathepsin D (~ 31 kDa), on the other hand, was more enriched in autolysosomes-containing MFs, while an intermediate form (~ 40 kDa) was more in LFs. We noted that the majority of SAP-C and SAPs were enriched in MFs comparing to WT controls (Fig. 8B). Noticeably, an increased SAP-C level, but a reduced mature cathepsin-D level, was found in 5xFAD MFs compared to WT controls. The enhanced delivery of SAP-C into lysosomes is expected to induce LMP and release of hydrolases from lysosomes as previously suggested [41–43]. Our results are in line with the notion that a reduced cathepsin-D level in MFs likely resulted from LMP-mediated release of cathepsin-D, triggered by an enhanced accumulation of SAP-C in autolysosomes/mature lysosomes. Consistently, we also noted an enhanced SAP-C protein level in LFs in 5-month-old 5xFAD mouse brains (Supplemental Fig. 6). Moreover, we found an increased level of galectin-3 (Gal-3), known as lysosomal damage sensor protein [46, 47], in LFs. This data also implies lysosomal damage in 5xFAD mouse brains associated with an enhanced lysosomal accumulation of SAP-C.

## Discussion

We have recently shown that DNs are enriched with heterogenous vesicles, growing sequentially in three layers in the surrounding amyloid plaque core: ATG9A-containing pre-autophagosomes are present in the inner layer (1st layer), RTN3-conaining clustered tubular ER is present in the middle layer (2nd layer), and multi-vesicles and mature autophagosomes labeled by RAB7 and LC3 are present in the outer layer (3rd layer) [11]. The lysosome-like multi-vesicle bodies have been previously shown to be accumulated in DNs and in glia cells [15, 31, 48, 49], but it was not clear when LAMP1-containing lysosomes are enriched in DNs. In this study, we demonstrate that DNs enriched lysosomes, containing LAMP1 and SAP-C, as early as amyloid plaques begin to form. With the growth of amyloid plaques, axonal degeneration near plaques is also increasing and functional lysosomes in axons are reduced, and this may viciously facilitate the growth of amyloid plaques. Thus, preventing lysosomal damage in axons is likely to be an effective intervention strategy to reduce amyloid toxicity.

Lysosomes are highly dynamic subcellular organelles originating from the Golgi. They acquire hydrolases through a series of dynamic events during lysosomal biogenesis [50, 51]. In neurons, lysosomes are distributed not only in the soma, but also in axons and dendrites [38, 52–54]. LAMP1, a commonly used lysosomal marker, is localized on the membranes of endosomes after synthesis and is transferred to lysosomal organelles via the endo-lysosomal pathway. It has been shown that a significant pool of lysosomes, lacking degradation capacity due to devoid of certain hydrolases such as cathepsin-B, −D and glucocerebrosidase, is more enriched in axons and dendrites, while hydrolase-containing lysosomes are more restricted to neuronal soma [34, 55]. SAPs (SAP-A to -D) are small amphipathic glycoproteins that are processed from their precursor, PSAP, mostly in lysosomes, and act as activators of glucocerebrosidase (GCase) or galactocerebrosidase during lysosomal hydrolysis of glycosphingolipids [56, 57]. The functions of each SAP are specific to their associated hydrolases, and deficiency of one may not be compensated by another [58, 59]. Mutations in PSAP may cause deficiencies in full PSAP or in a specific SAP, which leads to an excessive accumulation of lysosomal sphingolipids in certain organs, specifically in spleen, liver, bone, and bone marrow, and are associated with numerous pathological conditions collectively termed as lipid storage disorders [56, 60, 61]. SAP-C is particularly required for activating GCase and is present in a large portion (~ 64%) of peripheral LAMP1^+^ vesicles [36]. Our results showed that hydrolase-deficient but SAP-C-enriched LAMP1^+^ primary lysosomes were accumulated in the 1st layer of DNs during the initial plaque formation stage (Figs. 2 and Fig. 3A-O). It is expected that the accumulation of numerous vesicles or multi-vesicle bodies in DNs surrounding amyloid plaques are likely proportional to a gradual impairment of axonal trafficking due to continuous Aβ accumulation in AD brains. This is because the hydrolase-enriched LAMP1^+^ lysosomes, viewed as degradative lysosomes and predominantly localized in neuronal soma, are anterogradely transported to distal axons to maintain local degradation capacity [38] and the autophagosomes formed at distal neurites can be retrogradely transported to somata for degradation [55, 62]. The early trapping of such lysosomes in DNs likely impairs this trafficking balance, various lysosomal functions including GCase and lipid metabolism.

However, at the initial plaque-forming stage, axonal trafficking may only be restrictively impaired to areas containing ATG9A^+^ vesicles or LAMP^+^/SAPs^+^ primary lysosomes, as active lysosomal transport along axons appears to be functional based on visible LAMP1-labelled lysosomes distributed along axons (Fig. 5A, supplemental Fig. 3, left panel). At this early stage, endo-lysosomes at distal axons can still be partially maintained and are transported from neuronal soma. At later stages of plaque growth, when amyloid loads are sufficiently high to block the trafficking of degradative lysosomes from soma, LAMP1^+^ lysosomes are rarely visible in neurofilament-positive axons (Fig. 5A, supplemental Fig. 3, right panel). Under these conditions, local degradation capability is likely diminished and non-degraded autophagosomes and endosomal vesicles at the axonal terminals form an outer layer of DNs surrounding plaques [11]. Hence, gradual deposition of Aβ during plaque growth appears to disrupt the balance between the primary and functional lysosomal pools in neurites by interrupting their dynamic trafficking.

Both protein levels and CSF levels of PSAP are elevated in AD brains [63, 64]. An enrichment of SAP-C in lysosomes induce lysosomal membrane permeability (LMP) and LMP-mediated apoptotic cell death [41–43]. We noted a significant reduction of mature cathepsin D levels in autolysosomes/mature lysosomes, correlated with more SAP-C in 2-month-old 5xFAD (Fig. 8A-C). While cathepsin D is the main protease for processing of PSAP into SAPs [65], an enhanced SAP-C, but reduced cathepsin D, in autolysosome-enriched fractions likely an event resulted from LMP-mediated release of hydrolases such as cathepsin D. SAP-C enrichment also likely causes the damage of lysosomal maturation as demonstrated by an increased level of a lysosomal damage sensor protein, galactin-3 in LFs of 5-month-old 5xFAD compared to WT (supplemental Fig. 6A-B). Thus, in addition to Aβ-induced disruption of lysosomal trafficking [31, 66], enhanced SAP-C protein levels and SAP-C accumulation in lysosomes potentially lead to LMP [67–70] and induction of LMP-mediated apoptosis [71–73], which eventually could lead to neuronal degeneration in AD brains. Further studies will be conducted to attest this postulation.

We noted that SAP-C^+^-DNs disappeared at older AD mouse brains and late stage of AD progression in human brain (Fig. 3 and Fig. 4). We speculated that lysosomal exocytosis in response to elevated calcium (Ca^2+^) levels during amyloid plaque growth could lead to a selective reduction in SAP- and LAMP1-labelled DNs in older AD mouse brains. Lysosomal exocytosis is a Ca^2+^-regulated process in which lysosomes are docked at and fuse with the plasma membrane through LAMP1, in which it releases the luminal contents out of cells [74, 75]. Dysregulation of Ca^2+^ signaling, perhaps related to the perturbed regulation of ER Ca^2+^ signaling, the impaired ability of mitochondria for Ca^2+^ buffering, and elevated levels of intracellular Ca^2+^ has been shown in AD brains [76–78]. The tubular domains of ER and mitochondria play a critical role in intracellular Ca^2+^ balancing [79, 80]. Thus, subsequent accumulation of dysfunctional tubular ER and clustered mitochondria in the 2nd layer of DNs, at relatively later time points [11, 12], may contribute to elevated intracellular Ca^2+^ levels in neurons; this, in turn, would stimulate the exocytosis of accumulated LAMP1- and SAP-labeled primary lysosomes, resulting in a gradual reduction in LAMP1^+^/SAPs^+^ DNs at later time points (i.e., 9-months; Figs. 3A-R) when tubular ER/mitochondria-mediated DNs (i.e., RIDNs) are extensively accumulated in surrounding Aβ plaques (Supplemental Fig. 2A). Future in vitro and in vivo studies measuring Ca^2+^ signaling and lysosomal exocytosis in cultured neurons and in AD brain may reveal the underlying mechanisms.

Intriguingly, our study also revealed that immunohistochemically-detectable LAMP1 near mature amyloid plaques appeared to be in DAM, rather than in DNs. We showed that LAMP1 was mainly detected in DAM after plaque formation is stabilized in AD mouse brains as well as in AD postmortem brains. At the same time, LAMP1 immunoreactivity in intact axons was decreased, likely due to severe axonal degeneration as well as loss of LAMP1-immuno-epitopes of damaged autolysosomes in AD DNs. Hence, caution should be used when interpreting LAMP1 in DNs at late plaque-forming stages. The detection of both LAMP1 and hydrolases in DAM may indicate that phagocytic DAM perhaps retain functional lysosomes at later stages of AD development, or even during clinical manifestation [3, 4]. Further functional assays will be needed in order to clarify the state of degradative lysosomes in DAM.

In summary, using three different AD mouse models and AD postmortem brain samples, we have shown that accumulated LAMP1^+^ DNs at the early plaque formation stage are mostly SAP-enriched, but hydrolase-deficient primary lysosomes, dissociate from plaques after plaque formation eventually reaching a plateau. Our data suggest that Aβ deposition will impair lysosomal biogenesis at the initial plaque-forming stage. Enhanced lysosomal accumulation of SAP-C could be a contributing factor to lysosomal dysfunction in AD brains by inducing leakages of lysosomal hydrolases in DNs. Dysfunctional lysosomes will also facilitate tau pathologies through tau secretion and propagation [81]. Hence, preventing lysosomal damage and stimulating lysosomal biogenesis at early pre-clinical phases is likely an alternative AD therapeutic strategy.

## Supplementary Information

**Additional file 1: Supplemental Fig. 1.** Absence of cathepsins and endosomal proteins in DNs. **(A)** The fixed brain sections from a 6-month-old APP^NL-G-F^ mouse were co-labelled either with LAMP1 and cathepsin B or cathepsin D. **(B)** The fixed brain sections from a 6-month-old APP^NL-G-F^ mouse were co-labelled with 6E10 and EEA1/ RAB11 or Aβ42 and RAB4A/ RAB6A antibodies. **(C)** AD postmortem brain sections were either triple stained with Thio-S, SAP-C and RTN3 antibodies. Cathepsins or endosomal proteins were not appeared in DNs in APP^NL-G-F^ mouse brains. **Supplemental Fig. 2**. LAMP1, SAPs, ATG9A and RTN3 are differentially enriched in different populations of DNs in various ages of AD mouse brains. (**A**) Fixed brain sections from 1.5-, 3, 6-, 9- and 15-month (m)-old APP^NL-G-F^ mice were co-stained either with LAMP1 and SAPs or LAMP1 and RTN3. SAPs- and RTN3-marked DNs appeared in similar time point, but LAMP1-marked DNs appeared early during plaque growth. **(B)** The fixed brain sections from 3- and 15-m-old APP^NL-G-F^, 2.5- and 10-m-old 5xFAD and 6- and 16-m-old PA mice were triple-stained with LAMP1, ATG9A and RTN3. **(C)** 15-m-old APP^NL-G-F^ mouse brain sections were co-stained with LAMP1 and cathepsin B or cathepsin D**.** LAMP1^+^-DNs is reduced in older AD mouse brains and it mostly labeled center of the plaque, which was colocalized with cathepsins. **Supplemental Fig. 3**. Disruption of plaque surrounding axonal network in older APP^NL-G-F^ mouse brains. The Fixed brain samples from 1.5- and 15-month (m)-old APP^NL-G-F^ mice were co-stained with LAMP1 and SMI31 antibodies. SMI31 labeled axons were interact and LAMP1 labelled lysosomes were visible (indicated by white arrowhead) in axons at younger age when plaque formation just started. The axonal integrity was destroyed, and lysosomes were not visible in plaque surrounding area at older age. **Supplemental Fig. 4**. LAMP1 is mostly localized in microglia in older AD mouse brains. **(A)** The brain sections from 2.5-, 4 and 10-month (m)- old 5xFAD mice were co-labelled with LAMP1 and IBA1 or SAMI31. **(B)** The fixed brain section from PA mice at 6 and 16 months were co-stained with LAMP1 and IBA1 antibodies. **(C)** The fixed brain samples from a 10-m-old 5xFAD, a 16-m-old PA and a 9-m-old APP^NL-G-F^ mouse were triple-stained with LAMP1, ATG9A and IBA1. **(D)** Fixed brain section of a 9-m-old APP^NL-G-F^ mouse were triple-stained with cathepsin-D, LAMP1 and IBA1. LAMP1 mostly colocalized with IBA1 and cathepsin D in older AD mouse brains. **Supplemental Fig. 5**. Changes of proteins levels in AD mouse brains. (**A)** The protein level of LAMP1, PSAP, SAP-C, SAPs, ATG9A and calnexin were detected in the cortex of each age group (m, month) of APP^NL-G-F^ mice using Western blotting. **(B)** The band intensity for each protein was measured using Fiji software and the average of each age group was calculated after normalizing with calnexin. **(C)** The protein level of LAMP1, PSAP, SAP-C, SAPs, ATG9A and calnexin were detected in the cortex of each age group of 5xFAD mice. **(D)** The band intensity for each protein was measured and normalized with calnexin. * indicate a significant difference at *P* ≤ 0.05. The level of LAMP1, SAPs and SAP-C were increased during plaque growth in AD mouse brains. **Supplemental Fig. 6.** Increased level of SAP-C and galectin-3 in 5xFAD lysosomes. Variable sized organelles were enriched in mitochondria (MF)- and lysosomal (LF)- fractions by sequential centrifugation. **(A)** MF and LF obtained from 5- month-old WT and 5xFAD mouse brains were subjected to Western blot analysis. **(B)** Band intensity for each protein was measured and standardized to GAPDH. * indicate significant differences at *P* < 0.05. The protein levels of SAP-C and a lysosomal damage censor protein galectin-3 (Gal-3) were increased in the 5xFAD LF fraction. **Supplemental Table 1**. The detail information of AD postmortem brain samples collected from NIH brain bank.

**Additional file 2.**

## Abbreviations

Aβ: β-amyloid
AD: Alzheimer’s disease
APP: Amyloid precursor proteins
ATG9A: Autophagy related protein 9A
DAB: 3,3′-Diaminobenzidine
DAM: Disease associated microglia
DNs: Dystrophic neurites
EEA1: Early endosome antigen 1
ER: Endoplasmic reticulum
FBS: Fetal bovine serum
FAD: Familial AD
Gal-3: Galectin-3
Gcase: β-Glucocerebrosidase
GFAP: Glial fibrillary acidic protein
IBA1: Ionized calcium-binding adapter 1
LAMP1/LAMP2: Lysosomal-associated membrane protein 1/2
LC3: Microtubule associated protein light chain 3
LMP: Lysosomal membrane permeabilization
LPL: Lipoprotein lipase
NF-L: Neurofilament-L
PBS: Phosphate buffer saline
PS: Presenile
PSAP: Prosaposin
RAB: Ras associated binding protein
RIDNs: RTN3-immunoreactive DNs
RTN3: Reticulon 3
SAP: Saposin
Thio-S: Thioflavin-S
WT: Wild type
