# Supplementary material for: Accumulation of saposin in dystrophic neurites is linked to impaired lysosomal functions in Alzheimer’s disease brains
Source: Mol Neurodegener. 2021 Jul 2;16:45. doi: 10.1186/s13024-021-00464-1 (PMC8254260; doi:10.1186/s13024-021-00464-1)
Supplement: Supplementary file 1 — Additional file 1: Supplemental Fig. 1. Absence of cathepsins and endosomal proteins in DNs. (A) The fixed brain sections from a 6-month-old APPNL-G-F mouse were co-labelled either with LAMP1 and cathepsin B or cathepsin D. (B) The fixed brain sections from a 6-month-old APPNL-G-F mouse were co-labelled with 6E10 and EEA1/ RAB11 or Aβ42 and RAB4A/ RAB6A antibodies. (C) AD postmortem brain sections were either triple stained with Thio-S, SAP-C and RTN3 antibodies. Cathepsins or endosomal proteins were not appeared in DNs in APPNL-G-F mouse brains. Supplemental Fig. 2. LAMP1, SAPs, ATG9A and RTN3 are differentially enriched in different populations of DNs in various ages of AD mouse brains. (A) Fixed brain sections from 1.5-, 3, 6-, 9- and 15-month (m)-old APPNL-G-F mice were co-stained either with LAMP1 and SAPs or LAMP1 and RTN3. SAPs- and RTN3-marked DNs appeared in similar time point, but LAMP1-marked DNs appeared early during plaque growth. (B) The fixed brain sections from 3- and 15-m-old APPNL-G-F, 2.5- and 10-m-old 5xFAD and 6- and 16-m-old PA mice were triple-stained with LAMP1, ATG9A and RTN3. (C) 15-m-old APPNL-G-F mouse brain sections were co-stained with LAMP1 and cathepsin B or cathepsin D. LAMP1+-DNs is reduced in older AD mouse brains and it mostly labeled center of the plaque, which was colocalized with cathepsins. Supplemental Fig. 3. Disruption of plaque surrounding axonal network in older APPNL-G-F mouse brains. The Fixed brain samples from 1.5- and 15-month (m)-old APPNL-G-F mice were co-stained with LAMP1 and SMI31 antibodies. SMI31 labeled axons were interact and LAMP1 labelled lysosomes were visible (indicated by white arrowhead) in axons at younger age when plaque formation just started. The axonal integrity was destroyed, and lysosomes were not visible in plaque surrounding area at older age. Supplemental Fig. 4. LAMP1 is mostly localized in microglia in older AD mouse brains. (A) The brain sections from 2.5-, 4 and 10-month (m)- old 5xFAD mi [file 13024_2021_464_MOESM1_ESM.docx]

**Supplemental Figures**

**
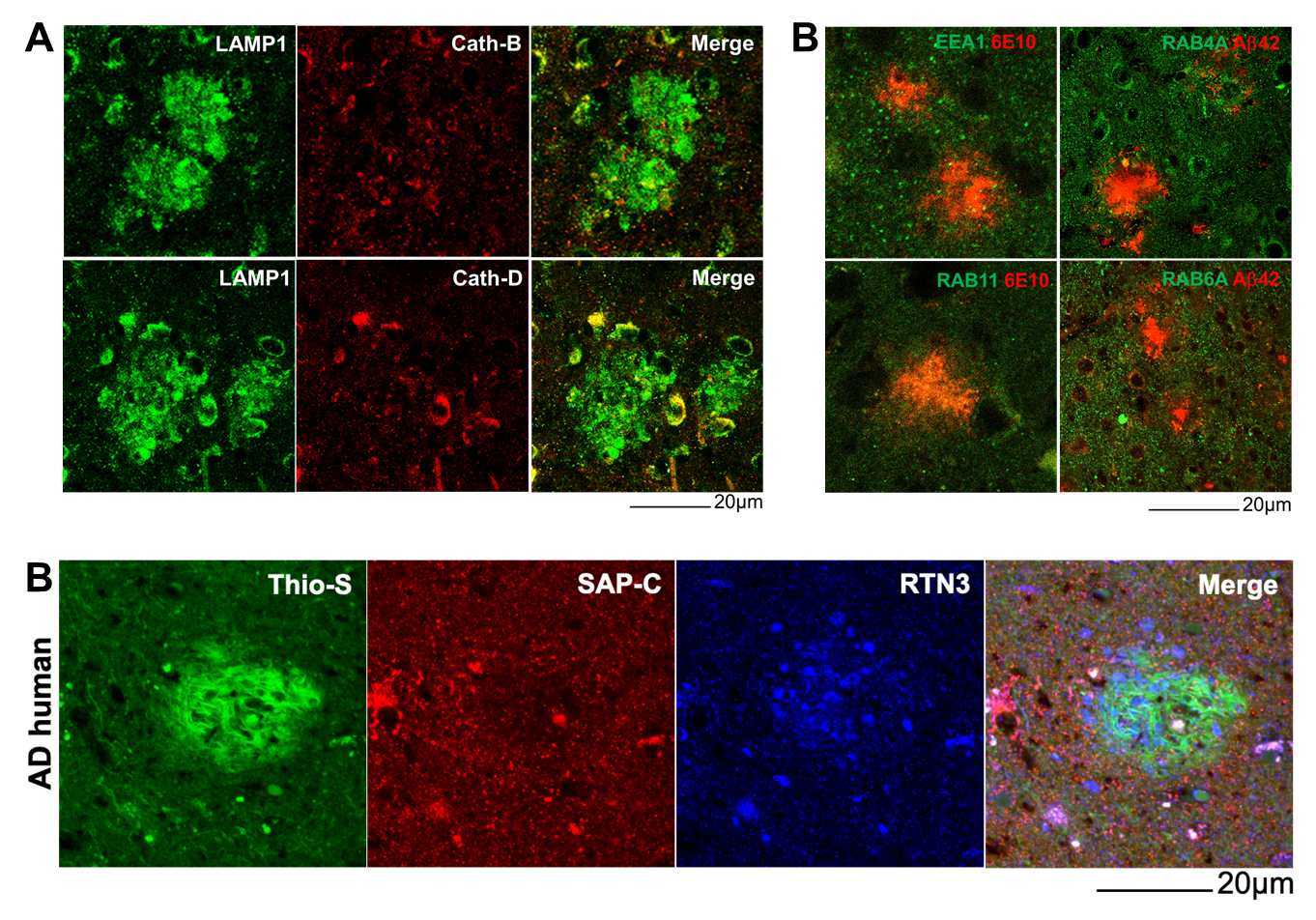
**

**Supplemental Figure 1: Absence of cathepsins and endosomal proteins in DNs. (A)** The fixed brain sections from a 6-month-old APP^NL-G-F^ mouse were co-labelled either with LAMP1 and cathepsin B or cathepsin D. **(B)** The fixed brain sections from a 6-month-old APP^NL-G-F^ mouse were co-labelled with 6E10 and EEA1/ RAB11 or Aβ42 and RAB4A/ RAB6A antibodies. **(C)** AD postmortem brain sections were either triple stained with Thio-S, SAP-C and RTN3 antibodies. Cathepsins or endosomal proteins were not appeared in DNs in APP^NL-G-F^ mouse brains.

**
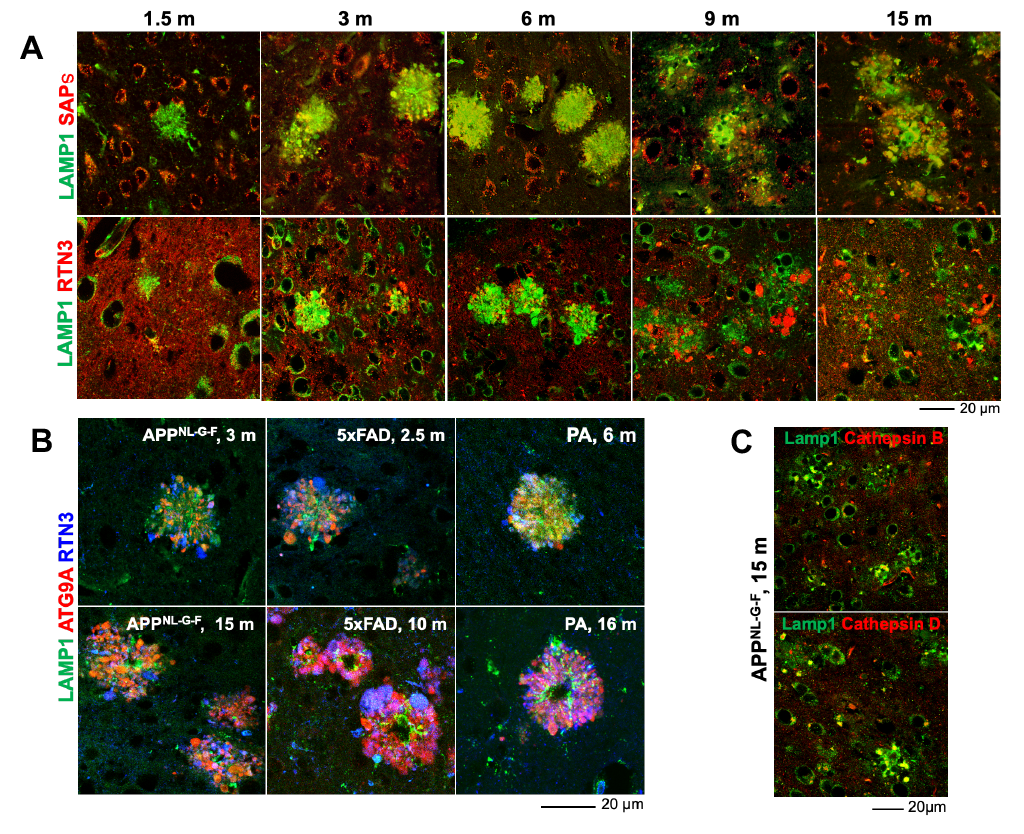
**

**Supplemental Figure 2: LAMP1, SAPs, ATG9A and RTN3 are differentially enriched in different populations of DNs in various ages of AD mouse brains.** (**A**) Fixed brain sections from 1.5-, 3, 6-, 9- and 15-month (m)-old APP^NL-G-F^ mice were co-stained either with LAMP1 and SAPs or LAMP1 and RTN3. SAPs- and RTN3-marked DNs appeared in similar time point, but LAMP1-marked DNs appeared early during plaque growth. **(B)** The fixed brain sections from 3- and 15-m-old APP^NL-G-F^, 2.5- and 10-m-old 5xFAD and 6- and 16-m-old PA mice were triple-stained with LAMP1, ATG9A and RTN3. **(C)** 15-m-old APP^NL-G-F^ mouse brain sections were co-stained with LAMP1 and cathepsin B or cathepsin D**.** LAMP1^+^-DNs is reduced in older AD mouse brains and it mostly labeled center of the plaque, which was colocalized with cathepsins.

**
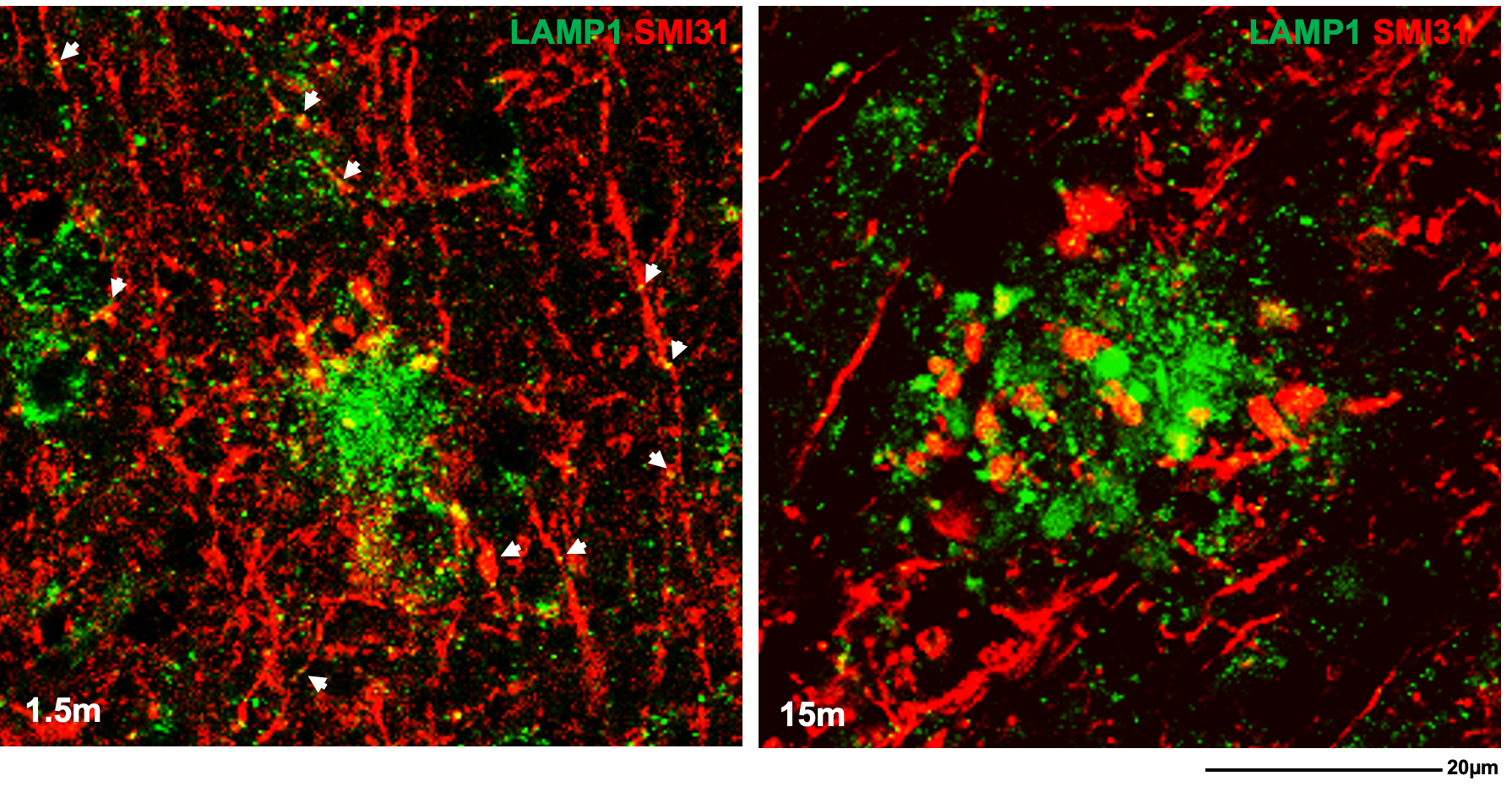
**

**Supplemental Figure 3: Disruption of plaque surrounding axonal network in older APP^NL-G-F^ mouse brains.** The Fixed brain samples from 1.5- and 15-month (m)-old APP^NL-G-F^ mice were co-stained with LAMP1 and SMI31 antibodies. SMI31 labeled axons were interact and LAMP1 labelled lysosomes were visible (indicated by white arrowhead) in axons at younger age when plaque formation just started. The axonal integrity was destroyed, and lysosomes were not visible in plaque surrounding area at older age.


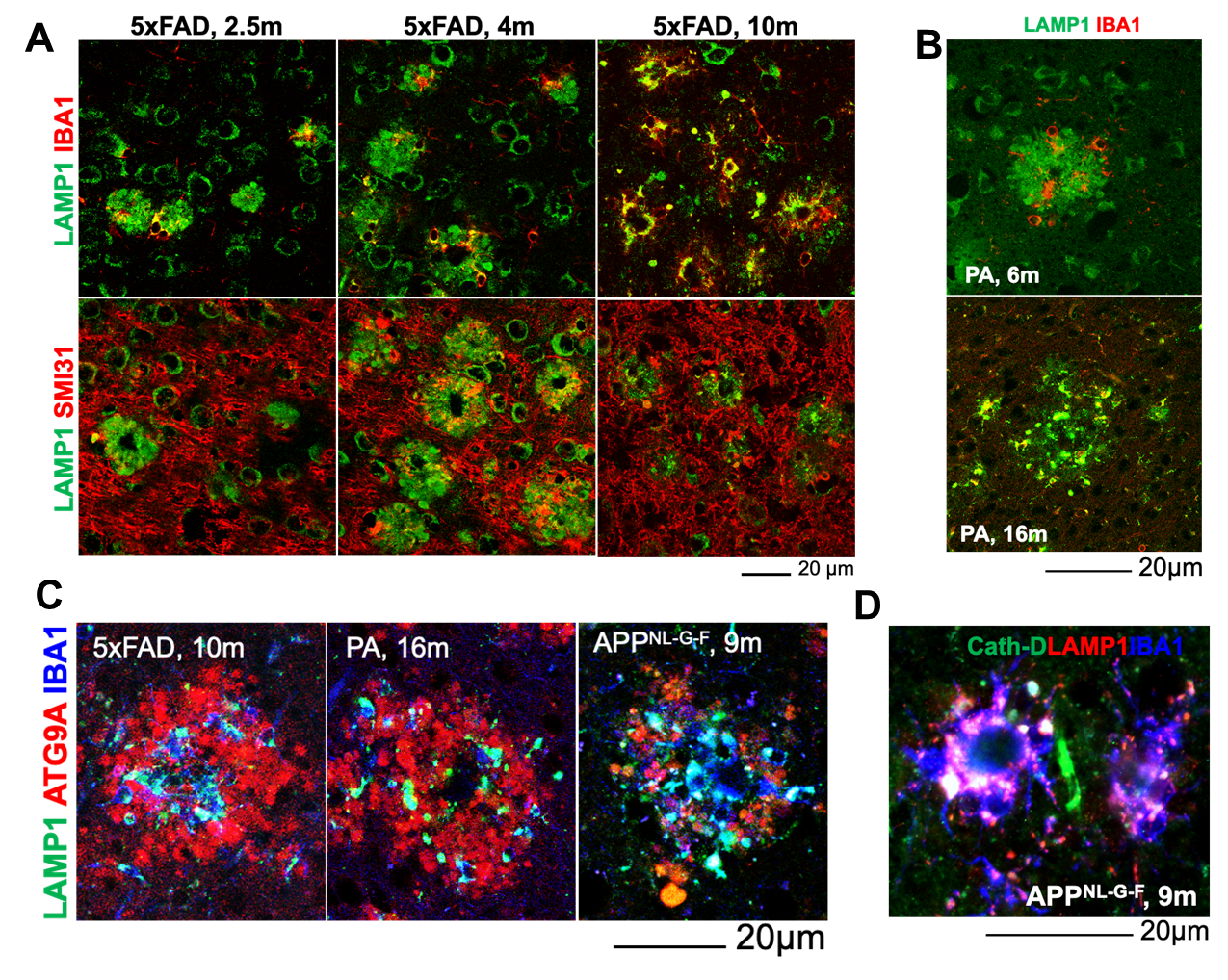


**Supplemental Figure 4: LAMP1 is mostly localized in microglia in older AD mouse brains. (A)** The brain sections from 2.5-, 4 and 10-month (m)- old 5xFAD mice were co-labelled with LAMP1 and IBA1 or SAMI31. **(B)** The fixed brain section from PA mice at 6 and 16 months were co-stained with LAMP1 and IBA1 antibodies. **(C)** The fixed brain samples from a 10-m-old 5xFAD, a 16-m-old PA and a 9-m-old APP^NL-G-F^ mouse were triple-stained with LAMP1, ATG9A and IBA1. **(D)** Fixed brain section of a 9-m-old APP^NL-G-F^ mouse were triple-stained with cathepsin-D, LAMP1 and IBA1. LAMP1 mostly colocalized with IBA1 and cathepsin D in older AD mouse brains.


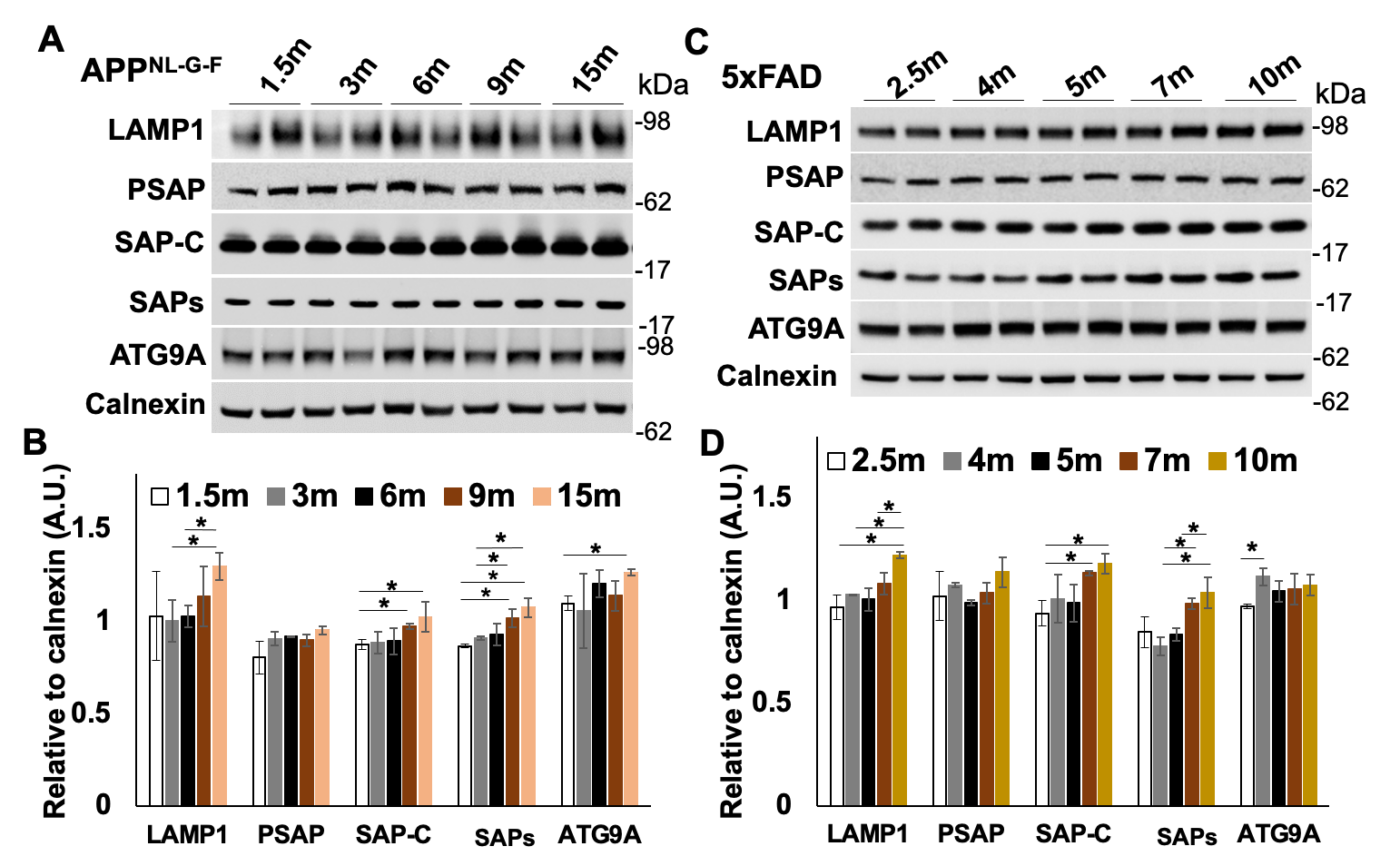


**Supplemental Figure 5: Changes of proteins levels in AD mouse brains. (A)** The protein level of LAMP1, PSAP, SAP-C, SAPs, ATG9A and calnexin were detected in the cortex of each age group (m, month) of APP^NL-G-F^ mice using Western blotting. **(B)** The band intensity for each protein was measured using Fiji software and the average of each age group was calculated after normalizing with calnexin. **(C)** The protein level of LAMP1, PSAP, SAP-C, SAPs, ATG9A and calnexin were detected in the cortex of each age group of 5xFAD mice. **(D)** The band intensity for each protein was measured and normalized with calnexin. * indicate a significant difference at P≤0.05. The level of LAMP1, SAPs and SAP-C were increased during plaque growth in AD mouse brains.

**Supplemental Figure 6: Increased level of SAP-C and galectin-3 in 5xFAD lysosomes.** Variable sized organelles were enriched in mitochondria (MF)- and lysosomal (LF)- fractions by sequential centrifugation. **(A)** MF and LF obtained from 5- month-old WT and 5xFAD mouse brains were subjected to Western blot analysis. **(B)** Band intensity for each protein was measured and standardized to GAPDH. * indicate significant differences at P<0.05. The protein levels of SAP-C and a lysosomal damage censor protein galectin-3 (Gal-3) were increased in the 5xFAD LF fraction.

**Supplemental Table 1: The detail information of AD postmortem brain samples collected from NIH brain bank.**

| **Sample#** | **Brain Bank/Source** | **Clinical diagnosis** | **Age at death** | **Sex** | **PMI** | **Neuropathology** | **B&B AD stage** | **Brain Region** |
| --- | --- | --- | --- | --- | --- | --- | --- | --- |
| 1 | Harvard | Non-demented | 78 | Female | 23.38 | 1. Atherosclerosis and arteriosclerosis.  2. Non-neuritic amyloid plaque.  3. Mild autolysis. | I | Hippocampus |
| 2 | Harvard | Non-demented | 90 | Male | 17.79 | 1. Small vessel cerebrovascular disease with atherosclerosis, arteriosclerosis, arteriolosclerosis, and infarcts and microinfarcts in cerebellar cortex, head of the caudate nucleus, precentral gyral white matter and prefrontal cortex.  2. Neurofibrillary degeneration with non-neuritic neocortical amyloid plaques. | I | Hippocampus |
| 3 | Harvard | Dementia, not specified | 70 | Female | 24.42 | 1. Neurofibrillary degeneration with non-neuritic neocortical amyloid plaques.  2. Atherosclerosis and arteriosclerosis | II | Hippocampus |
| 4 | Harvard | Dementia, not specified | 88 | Male | 15.58 | 1. Neurofibrillary degeneration with non-neuritic neocortical amyloid plaques.  2. Atherosclerosis and arteriosclerosis | II | Hippocampus |
| 5 | Harvard | Dementia, not specified | 86 | Female | 22.18 | 1. Severe amyloid pathology  2. Small vessel cerebrovascular disease with atherosclerosis, arteriosclerosis, arteriolosclerosis, a lacune in the caudate nucleus and microinfarcts in the trans-entorhinal cortex and in the amygdala. | V | Hippocampus |
| 6 | Harvard | Dementia, not specified | 80 | Male | 31 | 1. Severe amyloid pathology. 2. Cerebrovascular disease with atherosclerosis, arteriosclerosis, arteriolosclerosis, three examples of arterial mural necrosis, a resolved perivascular microhemorrhage in prefrontal cortex, a small recent hemorrhage infarct in the cerebellum, and multiple acute-early intermediate microinfarcts in the cerebral cortex and white matter. | V | Hippocampus |
| 7 | Harvard | Dementia, not specified | 72 | Female | 17.15 | 1. Amyloid angiopathy.  2. Atherosclerosis and arteriosclerosis | VI | Hippocampus |
| 8 | Harvard | Dementia, not specified | 61 | Male | 22.51 | 1. Amyloid angiopathy.  2. Arteriosclerosis | VI | Hippocampus |
| 9 | U of Kentucky | Dementia, not specified | 81 | Female | 8.10 | Severe amyloid pathology | VI | Hippocampus |
| 10 | U of Maryland | Progressive cognitive decline in multiple domains including executive, visuo-spatial, and memory. Suspicion of combined Alzheimer's disease and vascular dementia. | 82 | Female | 16 | Sever cerebral amyloid angiopathy | VI | Cerebral cortex |
| 11 | U of Maryland | Dementia, not specified | 80 | Male | 4 | Cerebral amyloid angiopathy with no corticobasal degeneration. |  | Cerebral cortex |
| 12 | Mount Sanai | Dementia, not specified | 69 | Female | 8.83 | Not specified; ApoE 3/3 | VI | Hippocampus |
| 13 | U of Maryland | Non-demented | 63 | Male | 17 | Not specified | Control | Hippocampus |
