# Supplementary material for: Accumulation of saposin in dystrophic neurites is linked to impaired lysosomal functions in Alzheimer’s disease brains
Source: Mol Neurodegener. 2021 Jul 2;16:45. doi: 10.1186/s13024-021-00464-1 (PMC8254260; doi:10.1186/s13024-021-00464-1)
Supplement: Supplementary file 2 — Additional file 2. [file 13024_2021_464_MOESM2_ESM.pptx]

## Slide 1
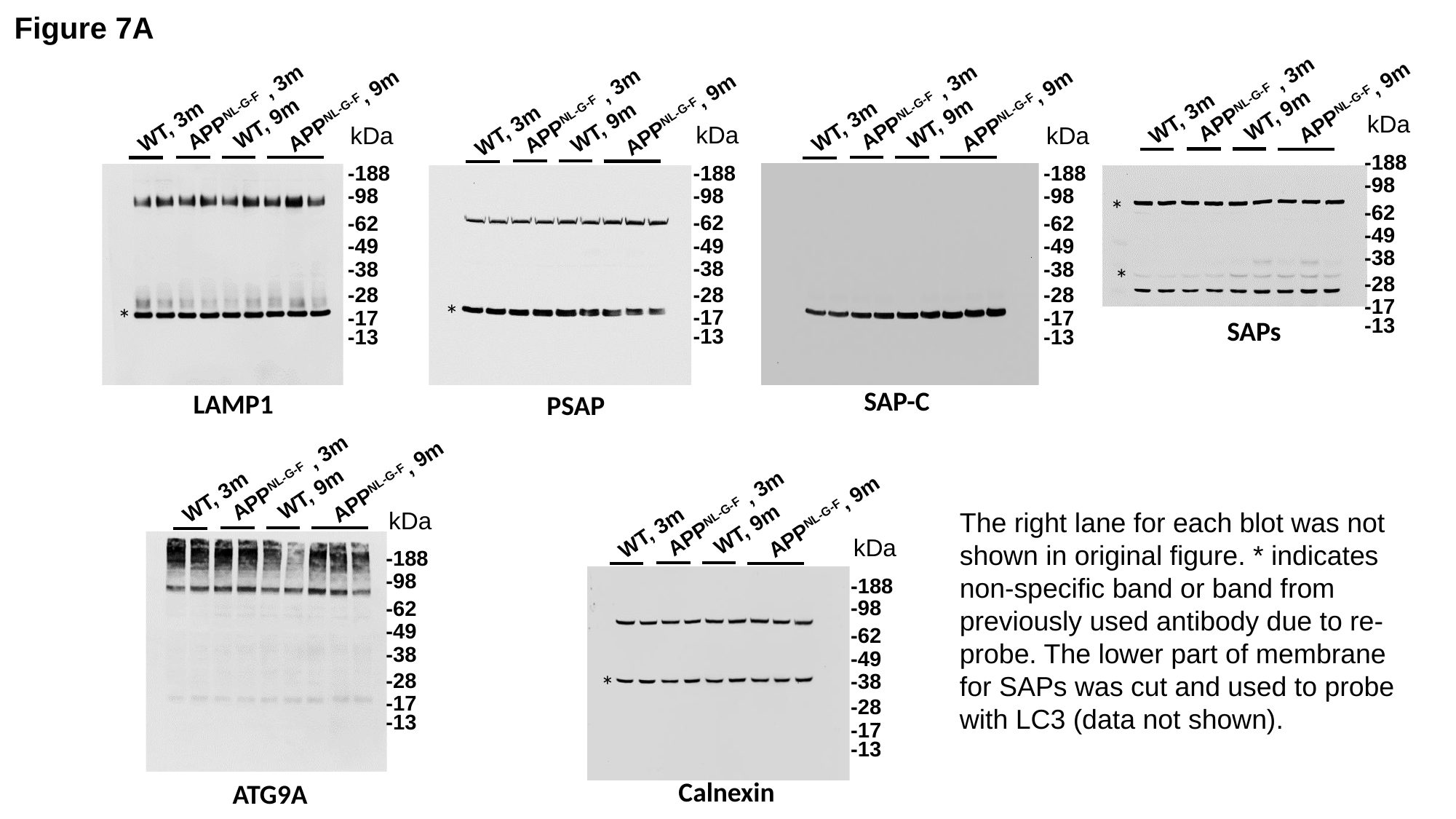

Figure 7A
APPNL-G-F , 3m
APPNL-G-F, 9m
WT, 9m
WT, 3m
APPNL-G-F , 3m
APPNL-G-F, 9m
WT, 9m
WT, 3m
APPNL-G-F , 3m
APPNL-G-F, 9m
WT, 9m
WT, 3m
APPNL-G-F , 3m
APPNL-G-F, 9m
WT, 9m
WT, 3m
kDa
-188
-98
-62
-49
-38
-28
-17
-13
kDa
-188
-98
-62
-49
-38
-28
-17
-13
kDa
-188
-98
-62
-49
-38
-28
-17
-13
kDa
-188
-98
-62
-49
-38
-28
-17
-13
*
*
*
*
SAPs
SAPs
SAP-C
LAMP1
PSAP
APPNL-G-F , 3m
APPNL-G-F, 9m
WT, 9m
WT, 3m
kDa
-188
-98
-62
-49
-38
-28
-17
-13
APPNL-G-F , 3m
APPNL-G-F, 9m
WT, 9m
WT, 3m
kDa
-188
-98
-62
-49
-38
-28
-17
-13
Calnexin
The right lane for each blot was not shown in original figure. * indicates non-specific band or band from previously used antibody due to re-probe. The lower part of membrane for SAPs was cut and used to probe with LC3 (data not shown).
*
ATG9A

## Slide 2
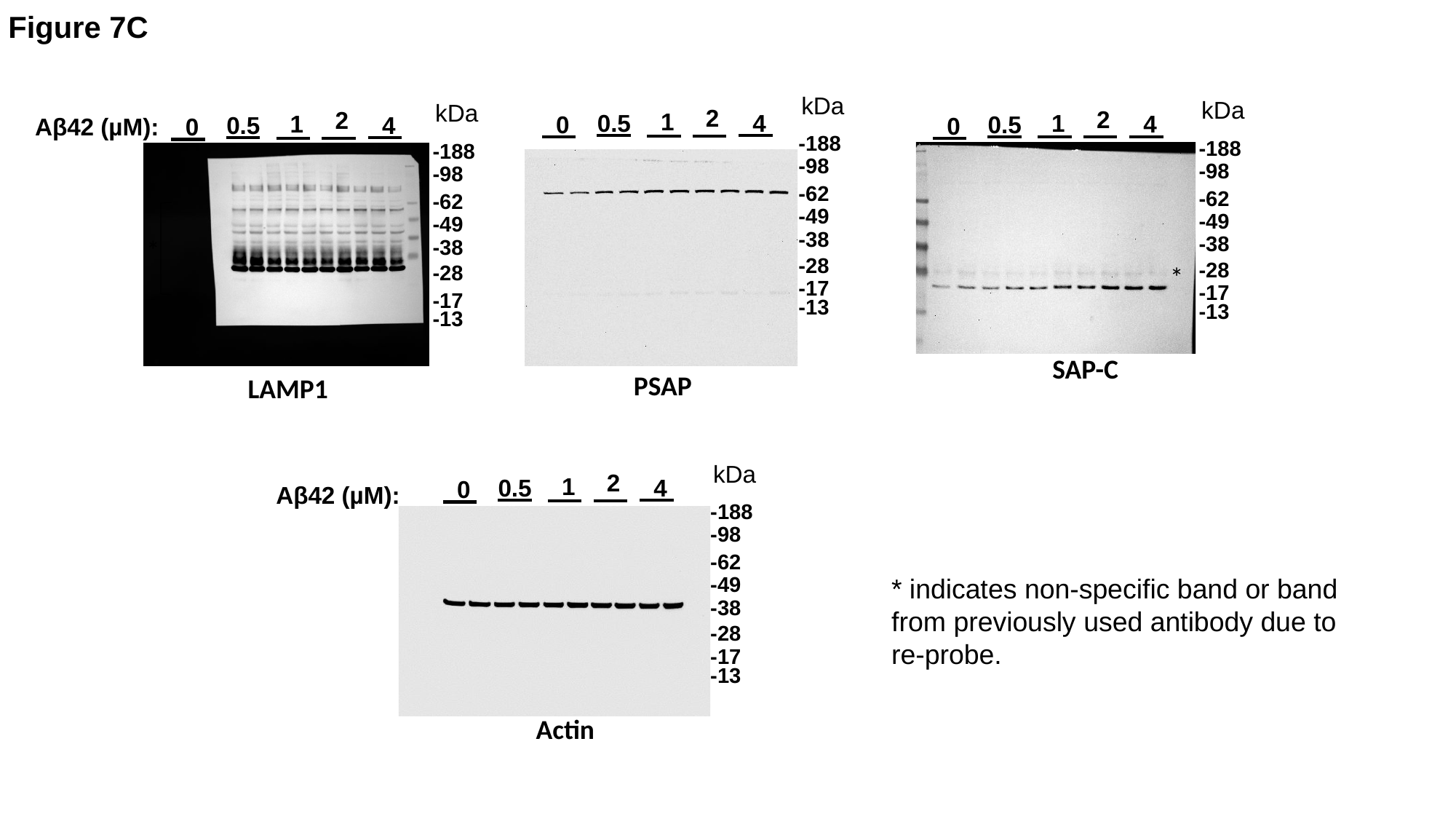

Figure 7C
kDa
-188
-98
-62
-49
-38
-28
-17
-13
2
1
4
0.5
0
PSAP
kDa
-188
-98
-62
-49
-38
-28
-17
-13
2
1
4
0.5
0
SAP-C
kDa
-188
-98
-62
-49
-38
-28
-17
-13
2
1
4
0.5
0
Aβ42 (µM):
LAMP1
*
*
kDa
-188
-98
-62
-49
-38
-28
-17
-13
2
1
4
0.5
0
Aβ42 (µM):
Actin
* indicates non-specific band or band from previously used antibody due to re-probe.

## Slide 3
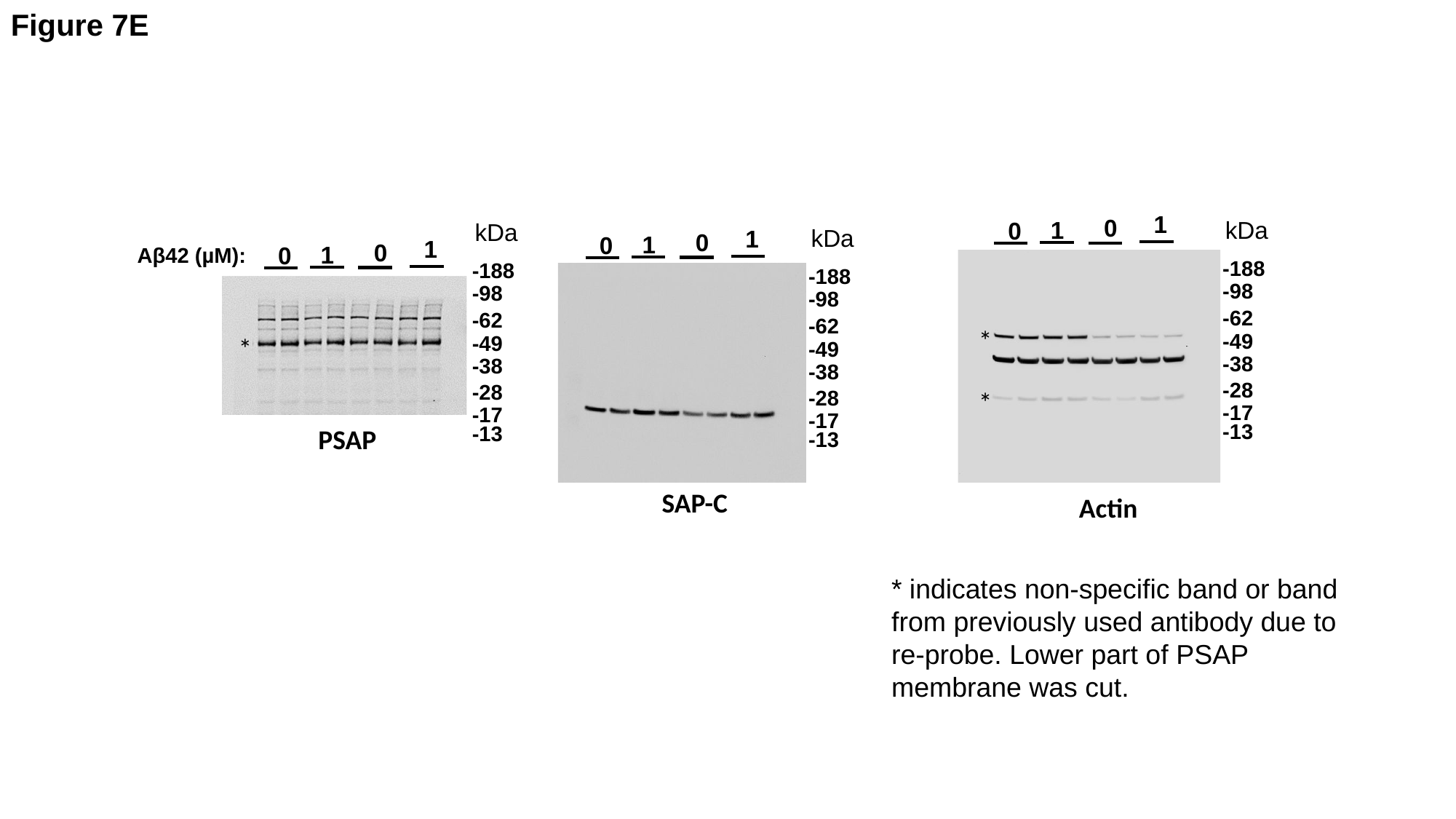

Figure 7E
1
0
1
0
kDa
-188
-98
-62
-49
-38
-28
-17
-13
kDa
-188
-98
-62
-49
-38
-28
-17
-13
kDa
-188
-98
-62
-49
-38
-28
-17
-13
1
0
1
0
1
0
1
0
Aβ42 (µM):
*
*
*
PSAP
SAP-C
Actin
* indicates non-specific band or band from previously used antibody due to re-probe. Lower part of PSAP membrane was cut.

## Slide 4
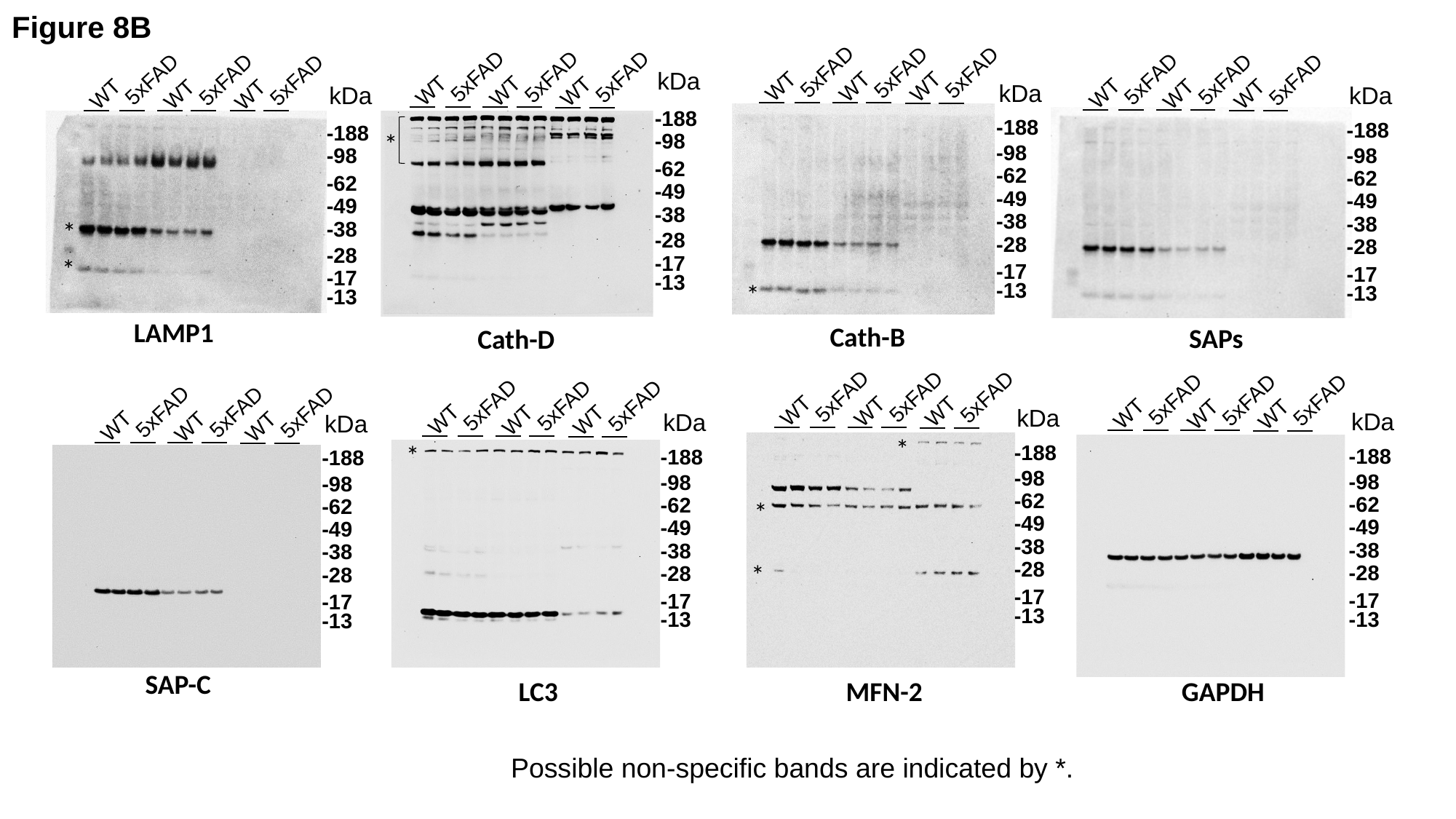

Figure 8B
5xFAD
5xFAD
5xFAD
WT
WT
WT
kDa
-188
-98
-62
-49
-38
-28
-17
-13
Cath-B
5xFAD
5xFAD
5xFAD
WT
WT
WT
kDa
-188
-98
-62
-49
-38
-28
-17
-13
Cath-D
5xFAD
5xFAD
5xFAD
WT
WT
WT
kDa
-188
-98
-62
-49
-38
-28
-17
-13
SAPs
5xFAD
5xFAD
5xFAD
WT
WT
WT
kDa
-188
-98
-62
-49
-38
-28
-17
-13
LAMP1
*
*
*
*
5xFAD
5xFAD
5xFAD
WT
WT
WT
kDa
-188
-98
-62
-49
-38
-28
-17
-13
MFN-2
5xFAD
5xFAD
5xFAD
WT
WT
WT
kDa
-188
-98
-62
-49
-38
-28
-17
-13
GAPDH
5xFAD
5xFAD
5xFAD
WT
WT
WT
kDa
-188
-98
-62
-49
-38
-28
-17
-13
LC3
5xFAD
5xFAD
5xFAD
WT
WT
WT
kDa
-188
-98
-62
-49
-38
-28
-17
-13
SAP-C
*
*
*
*
Possible non-specific bands are indicated by *.

## Slide 5
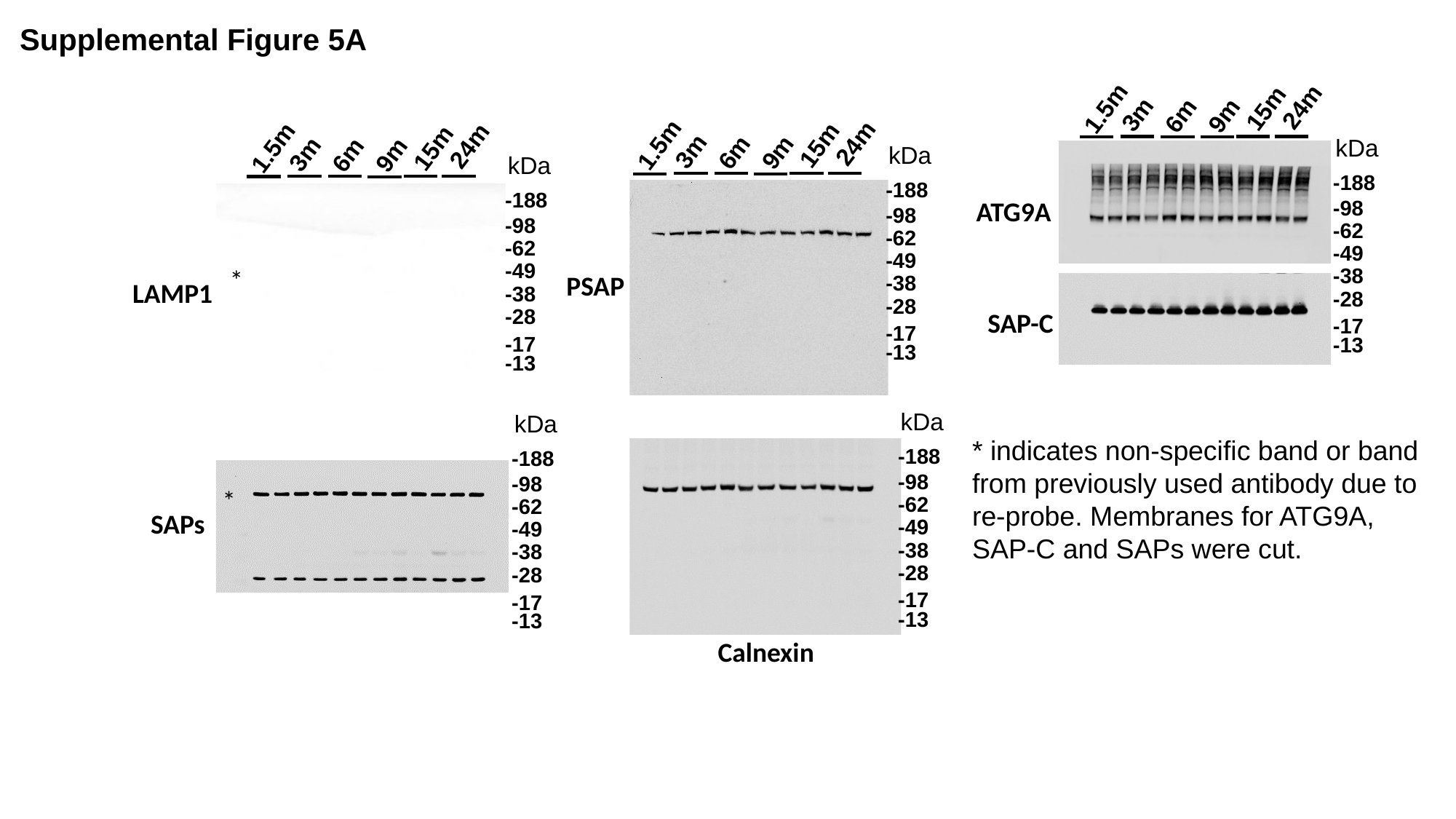

Supplemental Figure 5A
24m
15m
3m
6m
9m
1.5m
24m
15m
3m
6m
9m
1.5m
kDa
-188
-98
-62
-49
-38
-28
-17
-13
PSAP
24m
15m
3m
6m
9m
1.5m
kDa
-188
-98
-62
-49
-38
-28
-17
-13
LAMP1
kDa
-188
-98
-62
-49
-38
-28
-17
-13
ATG9A
*
SAP-C
kDa
-188
-98
-62
-49
-38
-28
-17
-13
Calnexin
kDa
-188
-98
-62
-49
-38
-28
-17
-13
SAPs
* indicates non-specific band or band from previously used antibody due to re-probe. Membranes for ATG9A, SAP-C and SAPs were cut.
*

## Slide 6
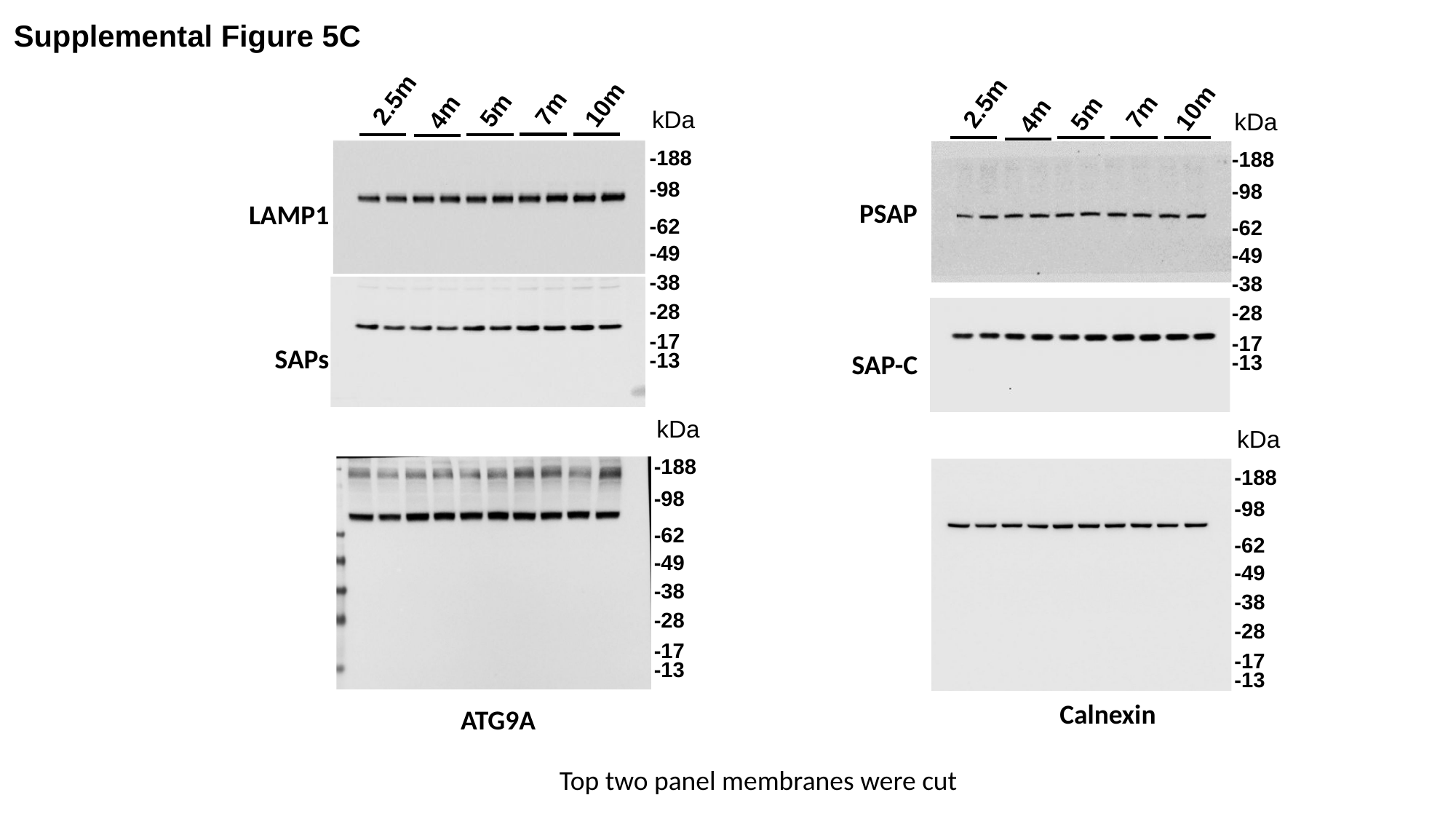

Supplemental Figure 5C
2.5m
7m
5m
10m
4m
kDa
-188
-98
-62
-49
-38
-28
-17
-13
LAMP1
SAPs
2.5m
7m
5m
10m
4m
kDa
-188
-98
-62
-49
-38
-28
-17
-13
PSAP
SAP-C
kDa
-188
-98
-62
-49
-38
-28
-17
-13
ATG9A
kDa
-188
-98
-62
-49
-38
-28
-17
-13
Calnexin
Top two panel membranes were cut
